# The Dilution Paradox in Extracellular Vesicle Flow Cytometry

**DOI:** 10.1002/jev2.70312

**Published:** 2026-06-23

**Authors:** Joyce Rops, Naomi Buntsma, Aleksandra Gąsecka, Leonie de Rond, Anne Yaël Nossent, Nyika D. Kruyt, Rienk Nieuwland, Edwin van der Pol

**Affiliations:** ^1^ Department of Laboratory Medicine, Laboratory Specialized Diagnostics & Research, Laboratory of Experimental Clinical Chemistry, Amsterdam UMC University of Amsterdam Amsterdam the Netherlands; ^2^ Biomedical Engineering & Physics Amsterdam UMC, University of Amsterdam Amsterdam the Netherlands; ^3^ Atherosclerosis and Ischemic Syndromes Amsterdam Cardiovascular Sciences Amsterdam the Netherlands; ^4^ Department of Neurology Leiden University Medical Center Leiden the Netherlands; ^5^ Department of Neurology Amsterdam University Medical Centers, University of Amsterdam Amsterdam the Netherlands; ^6^ 1st Chair and Department of Cardiology Medical University of Warsaw Warsaw Poland; ^7^ Department of Nutrition, Exercise and Sports University of Copenhagen Copenhagen Denmark; ^8^ Amsterdam Cardiovascular Sciences, Microcirculation Amsterdam the Netherlands; ^9^ Imaging and Biomarkers Cancer Center Amsterdam Amsterdam the Netherlands

**Keywords:** background, concentration measurement, dilution, extracellular vesicles, flow cytometry

## Abstract

Concentrations of extracellular vesicles (EVs) can be measured by flow cytometry, which detects fluorescence and light scattering signals from single particles. To ensure single particle detection, plasma samples are diluted based on their particle concentration, which can differ 100‐fold between samples. Here we studied how sample dilution affects EV detection by flow cytometry. EVs were measured in human plasma (i) with different concentrations of spiked‐in purified lipoproteins, (ii) at different dilutions, and (iii) using either scatter‐triggering or fluorescence‐triggering. In addition, the reliability of two published clinical EV studies was evaluated using an analytical model. Our results show that the minimum dilution factor to prevent swarm detection by the light scattering detector can exceed the maximum dilution factor that ensures labelled EVs outnumber fluorescent background events. This ‘dilution paradox’ precludes reliable detection of both fluorescence and light scattering signals of EVs for some samples. In the two clinical studies, 30% and 65% of the measurements were unreliable, depending on the label used. Therefore, results from previous studies should be re‐evaluated. The dilution paradox might be overcome by using fluorescence‐triggering at a fixed sample dilution, removing non‐EV particles from the sample, removing fluorescent particulates from staining reagents, and setting stricter inclusion criteria.

1

Definition of terms used throughout this article:

- Concentration ‐ The number of events detected by the flow cytometer divided by the measured sample volume, and multiplied by the dilution factor.
- Counts ‐ Number of events detected by the flow cytometer.
- Dilution factor ‐ Ratio of the final sample volume to the volume of the original (undiluted) sample.
- Dilution paradox ‐ Simultaneous need for a high dilution factor to prevent swarm detection by the light scattering detector and a low dilution factor to enable fluorescently labeled extracellular vesicles outnumbering fluorescent background events.
- Fuorescence‐triggering ‐ When the trigger threshold for data acquisition is set on a fluorescence detector of the flow cytometer.
- Fluorescent events ‐ Events whose fluorescence signal exceed the background level of the fluorescence detector and therefore fall within the applied fluorescence gate.
- Sample‐specific dilution factor ‐ Dilution factor that is specifically assigned to a sample in a measurement series to prevent swarm detection on the light scattering detector.
- Scatter‐triggering ‐ When the trigger threshold for data acquisition is set on a light scattering detector of the flow cytometer.
- Swarm detection ‐ Special form of coincidence detection where multiple particles, each generating a signal below the detection limit, are continuously and simultaneously illuminated by the laser beam of the flow cytometer. Their combined signal exceeds the trigger threshold, resulting in a falsely detected event.
- Total particle concentration ‐ Concentration of all particles in the sample volume that scatter sufficient light to exceed the trigger threshold of the light scattering detector. This may include extracellular vesicles, lipoproteins, protein aggregates, residual cells.

## 2 Introduction

Measuring the concentration of extracellular vesicles (EVs) in body fluids is challenging. One of the major challenges is the unique heterogeneity in size and biochemical composition of EVs, which can be quantified by single particle detection methods such as flow cytometry (Bettin et al., 2023). Flow cytometry detects fluorescence and light scattering signals from single particles flowing through focused laser beams. To measure these signals, the signal of at least one fluorescence or light scattering detector needs to exceed a predefined trigger threshold. Both fluorescence or light scattering detectors, or a combination thereof, can be used for triggering (Nolan and Duggan, 2018).

The detected concentration of total particles in plasma can differ more than 100‐fold between donors, when triggering on light scattering with a flow cytometer capable of detecting EVs with a diameter of ∼100 nm (Buntsma et al., 2023). To ensure single‐particle detection, that is, to prevent swarm detection, Buntsma et al. recommend customizing the dilution factor applied to each sample based on the total particle concentration of that sample (Buntsma et al., 2023). Thus, samples with higher total particle concentrations require larger dilution factors to prevent swarm detection than samples with lower total particle concentrations.

**FIGURE 1:**
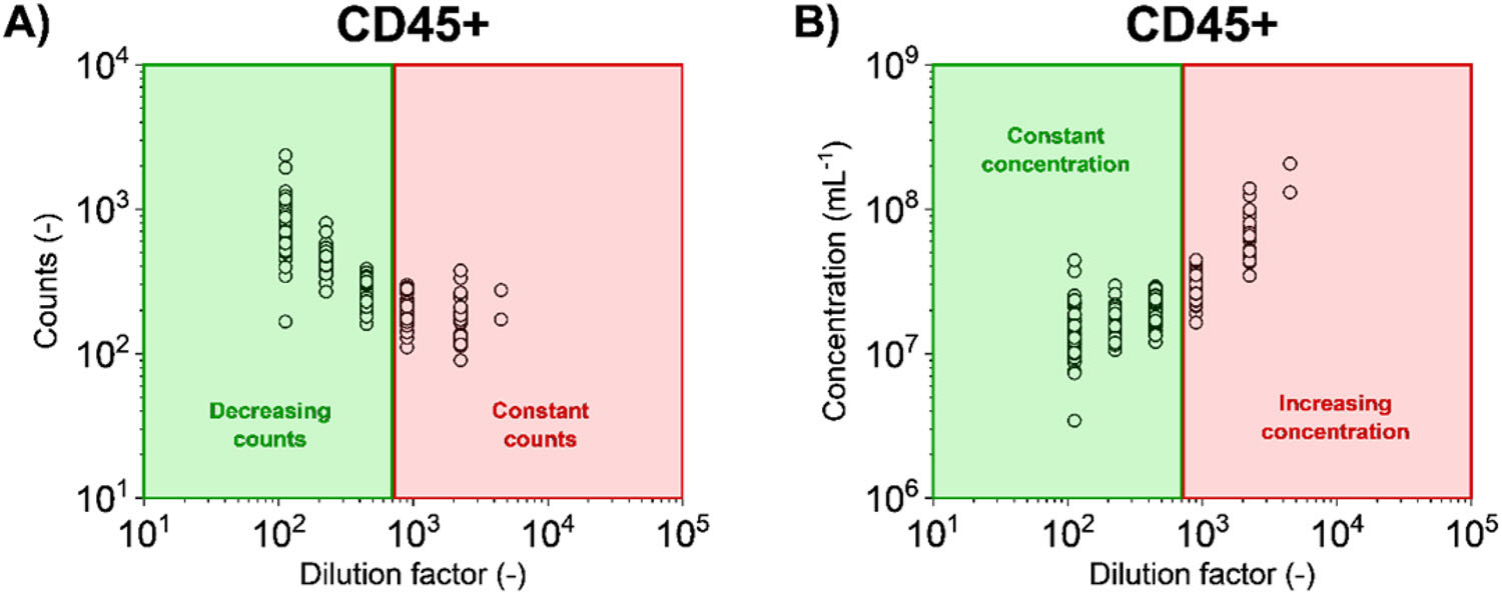
Sample‐specific dilution affects fluorescent particle concentration measurements in the clinical research study AFFECT EV (Gasecka et al., 2020). Data represent events that exceeded a side scattering cross‐section of 10 nm^2^ with diameter <1000 nm and positive (> 91 MESF) for the antibody label CD45‐APC (leukocyte‐marker). (A) Measured number of CD45+ events versus the dilution factor, showing a decrease in CD45+ events until a dilution factor of ∼10^3^, where the CD45+ events level off. (B) Concentration of CD45+ events calculated from the measured number of CD45+ events shown in A, versus dilution factor. From a dilution factor of ∼10^3^ the concentration of CD45+ events increases with the dilution factor. APC, allophycocyanin; MESF, molecules of equivalent soluble fluorochrome.

An example of the effect of sample‐specific dilution on EV concentrations is shown in Figure 1. Figure 1 shows data from the Antiplatelet Therapy Effect on Extracellular Vesicles (AFFECT EV) study (Gasecka et al., 2020), which utilized scatter‐triggered flow cytometry. As the plasma samples differed 100‐fold in total particle concentration, the applied dilution factor increased with increasing total particle concentration to prevent swarm detection. Figure 1A shows an inverse correlation between the detected total antibody‐positive particle number (dTA+PN) of CD45 and the sample‐specific dilution factor. This inverse correlation is observed up to a dilution factor of ∼10^3^. When the dilution factor exceeds 10^3^, the inverse relationship between the dTA+PN and the dilution factor is lost, and the dTA+PN remains constant.

Figure 1B shows the calculated total antibody‐positive particle concentration (cTA+PC) for CD45. This concentration is obtained by dividing the dTA+PN for CD45 (Figure 1A) by the measured sample volume, and multiplying the outcome with the applied dilution factor. In Figure 1B, the cTA+PC is constant up to a dilution factor of ∼10^3^, but increases at higher dilution factors. This increase is unexpected, because it suggests a correlation between the concentration of CD45+ EVs and the total particle concentration, while such a correlation neither seems biologically plausible nor has been observed before. As an alternative explanation, the CD45+ events at dilution factor > 10^3^ may not represent EVs but rather fluorescent background events.

Possible sources of these background events include antibody aggregates, instrumental noise and sample carryover. As fluorescent background events and EVs have overlapping signals that cannot be distinguished, EV counts must dominate the background counts to provide a meaningful estimate of the EV concentration. However, samples with relatively high total particle concentration require substantial dilution, which reduces the EV counts to a level approaching the fluorescent background counts. When fluorescent background events are multiplied by the dilution factor during calculation of the concentration, increasing dilution factors lead to increasingly overestimated concentrations of labelled EVs. Thus, samples have a maximum dilution factor yielding reliable concentrations of labelled EVs. However, this maximum dilution factor might be below the minimum dilution factor to prevent swarm detection by the light scattering detector. When this happens, the light scattering signals of these EVs cannot be reliably detected, because there is no dilution factor that (i) prevents swarm detection, and (ii) ensures the number of labelled EVs exceeds the number of fluorescent background events. We refer to such conflicting dilution requirements as the ‘dilution paradox’.

In this study, we hypothesize that sample‐specific dilution may result in overestimation of the EV concentration in samples with a high total particle concentration. To investigate our hypothesis, (i) we determined the effect of sample‐specific dilution on the calculated concentration of labelled EVs, (ii) evaluated two strategies enabling reproducible and comparable concentration measurements using either scatter‐ or fluorescence‐triggering, (iii) present a method that can be used to estimate the reliability of EV concentration measurements retrospectively. Together, this study provides recommendations on how to overcome the dilution paradox in EV flow cytometry measurements.

## 3 Methods

**FIGURE 2:**
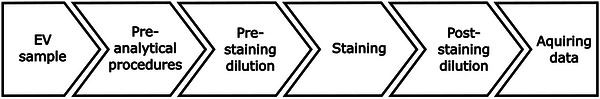
Schematic overview of the analytical workflow of a flow cytometry measurement to characterize extracellular vesicles (EVs). The pre‐ and post‐staining dilution factors, when multiplied, represent the dilution factor that is meant throughout this article.

Figure 2 shows the workflow of a typical flow cytometry measurement to characterize EVs in a biological sample. In the following sections each element in this schematic will be described.

### 3.1 Pre‐Analytical Procedures

Blood collection was performed according to the guidelines of the medical ethical committee of the Amsterdam University Medical Centre, location University of Amsterdam (WP22‐243 #22.298). Blood was collected from 20 healthy volunteers, 10 men (median age: 34 (20–64 years) and 10 women (median age: 33 (25–63) years) in Ethylene Diamine Tetra‐acetic Acid (Vacutainer EDTA, Becton Dickinson, Franklin Lanes, NJ) using a 21G needle (Elipse, Becton Dickinson, Franklin Lanes, NJ). Blood was centrifuged for 15 mins at 2500 g at room temperature without brake (Rotina 380R, Hettich, Tuttlingen, Germany). Plasma was collected to 10 mm above the buffycoat, transferred to a clean tube and centrifuged again using the same settings as before. Plasma was collected to 10 mm above the cell pellet. Plasma samples from all donors were pooled and aliquots of 100 µL were snap frozen in liquid nitrogen and stored at −80°C until use. Plasma was thawed in a water bath at 37°C for approximately 30 s prior to use. The pooled plasma was used in all experiments described in this article.

### 3.2 Dilution and Staining

**FIGURE 3:**
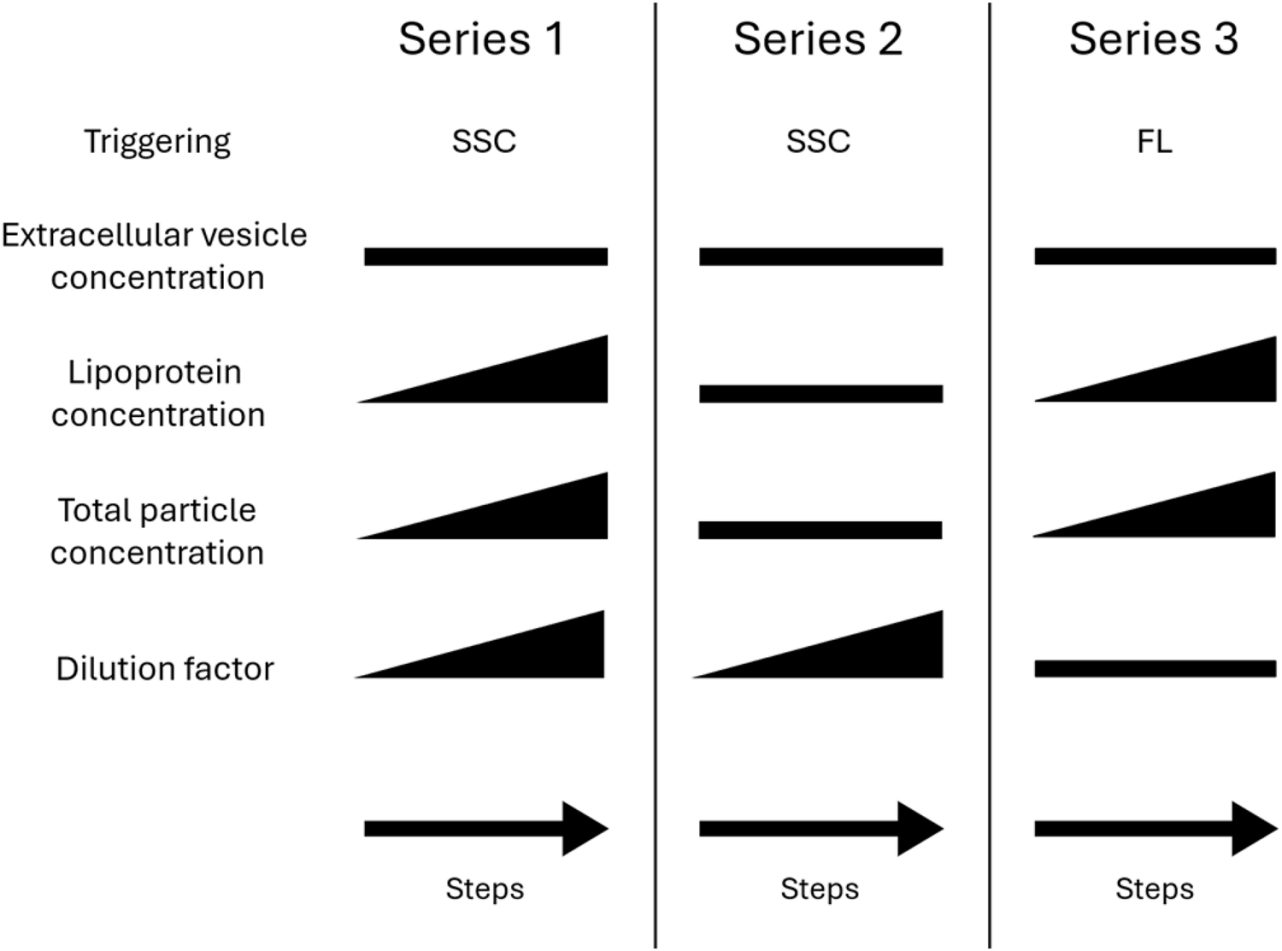
Overview of the three series of test samples that were prepared and measured by flow cytometry. The starting material for the sample series was pooled plasma from healthy donors. Triggering indicates whether the series was measured using side‐scatter (SSC) triggering or fluorescence (FL) triggering. Series 1: This sample series is used to illustrate the dilution paradox by determining effect of dilution on the calculated concentration of fluorescent events. By adding increasing concentrations of lipoproteins to plasma, the total particle concentration increases. As this series was measured using scatter‐triggering, the required dilution factor to prevent swarm detection increases with the total particle concentration. Series 2: This series is used to provide a possible solution to avoid the dilution paradox and show how repeatable and comparable concentration measurements can be performed. The initial extracellular vesicle‐, lipoprotein‐ and total particle concentrations remain constant throughout the series. The dilution factor that is applied before measurement increases with each step. This series was measured using scatter‐triggering. Series 3: With this series an alternative solution is explored using fluorescence‐triggering. As fluorescence‐triggering only detects fluorescent events, the required dilution factor is independent from the total particle concentration. Therefore, in this series the dilution factor remains constant. Increasing concentrations of lipoproteins are added to plasma, thereby increasing the total particle concentration.

Figure 3 shows an overview of the three series of test samples that were prepared to study the dilution paradox. Each series consists of a number of sequential samples in which one variable was varied. Details about the dilution and staining of these series of test samples will be discussed in the following sections.

#### 3.2.1 Series 1

Pooled plasma was prepared as described before. Very‐low‐density lipoprotein (VLDL) (cat. 437647, lot. D00159801, Calbiochem, EMD Chemicals Inc., San Diego, CA) and chylomicrons (lot. XA2201‐L, cat. CMIC15‐N, Alpha Diagnostics International Inc., San Antonio, TX) were mixed in a 1:1 concentration ratio. The lipoprotein mixture was added to the plasma. The total particle concentration increased from 7.3 × 10^10^ mL^−1^ (baseline, plasma, no lipoproteins added) to 1.0 × 10^14^ mL^−1^ in 8 steps leading to a $\sqrt{10}$ increase of the total particle concentration per step. Due to the increase in total particle concentration, also the dilution factor increases to prevent swarm detection (see Series 1 in Figure 3). To achieve this, the pre‐staining dilution factor was varied between 4.5 × 10^1^ and 6.4 × 10^4^.

20 µL of pre‐diluted samples incubated for 2 h at room temperature with 2.5 µL pre‐diluted CD45‐APC (B272158, Biolegend, San Diego, CA, final concentration 1.5 µg/mL) and 2.5 µL pre‐diluted CD62p‐FITC (cat. A07790, lot. 200042, CLB‐Thromb/6, Beckman Coulter, Brea, CA; final concentration 8.3 µg/mL). A post‐staining dilution of 11.25 was applied by adding 200 µL of Dulbecco's Phosphate Buffered Saline (DPBS) to the stained samples. The samples were measured in 4 replicates using flow cytometry with scatter‐triggering. Data are presented as dTA+PN (counts) versus dilution factor.

#### 3.2.2 Series 2

For Series 2, pooled plasma was serially diluted in DPBS in 8 steps of $\sqrt[{3}]{10}$ (Series 2 in Figure 3). To achieve this, the pre‐staining dilution factor was varied between 4.5·× 10^1^ and 8.9·× 10^3^. Staining and post‐staining dilution were identical to the method described for Series 1. After post‐staining dilution, the samples were measured in 8 replicates using flow cytometry with scatter‐triggering. Data are presented as dTA+PN (counts) versus dilution factor.

#### 3.2.3 Series 3

In this experiment, we prepared samples with increasing total particle concentration by adding VLDL and chylomicrons to pooled plasma, similar as described for Series 1. In Series 3, however, all prepared spike‐in samples (i.e. with increasing total particle concentration) were diluted 500‐fold in DPBS, see Figure 3. Lipoproteins were spiked‐in so that the total particle concentration ranged from 7.3 × 10^10^ mL^−1^ (baseline, no lipoproteins added) to 1.00 × 10^13^ mL^−1^ in 6 steps of $\sqrt{10}$. The total dilution factor was kept constant. 20 µL of pre‐diluted spiked samples were incubated for 2 h at room temperature with 2.5 µL of CD45‐APC and 2.5 µL of DPBS. A post‐staining dilution of 11.25 was applied by adding 200 µL of DPBS to the stained samples. After post‐staining dilution, measurements were performed using flow cytometry with fluorescence‐triggering.

### 3.3 Acquiring Data

Flow cytometry measurements were performed using an Apogee A60‐Micro (Apogee Flow Systems). Samples were prepared as described before and measured for 120 s at a flow rate of 3.01 µL/min. When scatter‐triggering was used, the trigger threshold was put on a value corresponding to a side‐scattering cross section of 3 nm^2^. When fluorescence‐triggering was used, a trigger threshold of 32 allophycocyanin (APC) molecules of equivalent soluble fluorochrome (MESF) was set on the APC fluorescence channel.

Details about instrument configuration and calibration, operating conditions, data acquisition and assay controls can be found in the MIFlowCyt‐EV in the Supplementary Material.

### 3.4 Characterization of the Effect of Sample‐Specific Dilutions on the Calculated Concentration of Fluorescent Events

The relationship between the number of measured fluorescent events $N_{F,M}$ and the dilution factor DF can be described as follows:

(1) $$N_{F,M}=\frac{N_{F,S}}{DF}+B,$$

where, $N_{F,S}$ is the number of fluorescent events in the undiluted sample and B is the number of fluorescent background events. To estimate the values of $N_{F,S}$ and B, the values of $N_{F,M}$ were first transformed using a 10‐base logarithm. The log_10_‐transformed data were then fitted to the function $\log_{10}\left(\frac{N_{F,S}}{DF}+B\right)$ using orthogonal distance regression.

The calculated concentration of fluorescent events $C_{F,C}$ can be described as follows:

(2) $$C_{F,C}=\frac{N_{F,S}}{V}+\frac{B\cdot DF}{V},$$

where, V is the measured sample volume and $N_{F,S}$ and B are obtained from Equation (1).

To study the dilution paradox, it is important to first establish the lower detection limit for the number of fluorescent events that can be reliably measured. Metrologically traceable reference materials do not yet exist for EV concentration measurements. Therefore, classic methods to determine the assay detection limits, like calibration curves, cannot be applied. Below we describe how the values found in Equation (1) can be used to characterize the assay detection limits. The limit of detection (LoD) for fluorescent events was defined as the lowest number of fluorescent events that can be distinguished from the background event level:

(3) $$\mathrm{Lo}D_{N}=B+3.3*SE_{B},$$

where, $SE_{B}$ is the standard error of the background counts. The concentration of fluorescent events in the undiluted sample can be estimated using the measured sample volume V as follows:

(4) $$C_{F,S}=\frac{N_{F,S}}{V}\;\;\pm \;\frac{3.3\cdot SE_{N_{F,S}}}{V},$$

In Equations (3) and (4) we chose to assign an uncertainty of 3.3 times the standard error based on the 95% confidence interval of zero false‐positives (*α* = 0.05) in combination with the 95% confidence interval of zero false‐negatives (*β* = 0.05). As the number of events is a discrete quantity, the value of $LoD_{N}$ was rounded up to the nearest integer.

Equations (1)–(4) are used to quantify the results of the experiments using the test sample series 1–3.

### 3.5 Retrospective Evaluation of Reliability of Clinical Study Results

Reliability of results from published studies that used sample‐specific dilution may be impaired due to the dilution paradox. Here, a method will be developed to retrospectively evaluate the reliability of previously published studies, using data from two clinical studies that were performed in our laboratory.

#### 3.5.1 AFFECT EV

AFFECT EV was an investigator‐initiated, single‐centre, randomized controlled trial to compare the effects of ticagrelor and clopidogrel, two drugs that inhibit platelet activation, on plasma concentrations of EVs in patients after acute myocardial infarction. Flow cytometry measurements were performed using an Apogee A60‐Micro. The study was conducted at the First Chair and Department of Cardiology, Medical University of Warsaw, Poland, in collaboration with the Amsterdam Vesicle Centre, Amsterdam University Medical Centres, The Netherlands. The study protocol was compliant with the Declaration of Helsinki and was approved by the Ethics Committee of the Medical University of Warsaw (approval number: KB/112/2016). The study was registered in the ClinicalTrials database under registration number NCT02931045. All details of this study have been published previously (Gasecka et al., 2020). Here only the flow cytometry data from AFFECT EV will be used.

#### 3.5.2 CINTICS

The observational clinical trial Circulating Nanotraces to Identify the Cause of Stroke (CINTICS) aimed to compare plasma concentrations of specific EVs in confirmed acute ischemic stroke patients with patients suspected to have acute ischemic stroke that appeared not to have ischemic stroke after further work‐up in the hospital. Flow cytometry measurements were performed using a Northern Lights (Cytek Biosciences). Patient inclusion took place at the emergency department of the Amsterdam University Medical Centres, location Academic Medical Centre. The protocol with deferred consent process was approved by the Ethical Review Board of the Amsterdam University Medical Centres, location Academic Medical Centre under approval number NL72929.018.20. All details of this study have been published previously (Buntsma et al., 2025). Here, only the flow cytometry data from CINTICS will be used. This study will hereafter be referred to as CINTICS.

#### 3.5.3 Retrospective Analysis

Data from AFFECT EV and CINTICS was represented as the number of fluorescent events versus dilution factor. For both studies, Equation (1) was fitted as described before to the flow cytometry data of each individual marker. Equation (3) was then used to calculate $LoD_{N}$ retrospectively.

A maximum reliable dilution factor could be defined as well. $DF_{max}$ is set as the highest dilution factor at which fluorescent particles from the sample can be distinguished from the $LoD_{N}$:

(5) $$DF_{max}=\frac{N_{F,S}}{LoD_{N}}\;,\;$$

Based on Equations (3) and (5), fluorescent particles can be distinguished from background in measurements where the number of fluorescent particles > $LoD_{N}$ and where dilution factor < $DF_{max}$. The measurements that fulfil the aforementioned criteria contain more fluorescent events associated with the sample than background and therefore are considered reliable. For each study, the percentage of reliable datapoints was estimated per label.

### 3.6 Data Analysis

Custom built software (MATLAB R2020b, MathWorks) was used to automate data calibration and processing of flow cytometry data. Graphing, data fitting and statistical analyses were performed using Origin Pro 2024b (OriginLab Corporation).

## 4 Results

### 4.1 Sample‐Specific Dilution Affects Concentration Measurements

**FIGURE 4:**
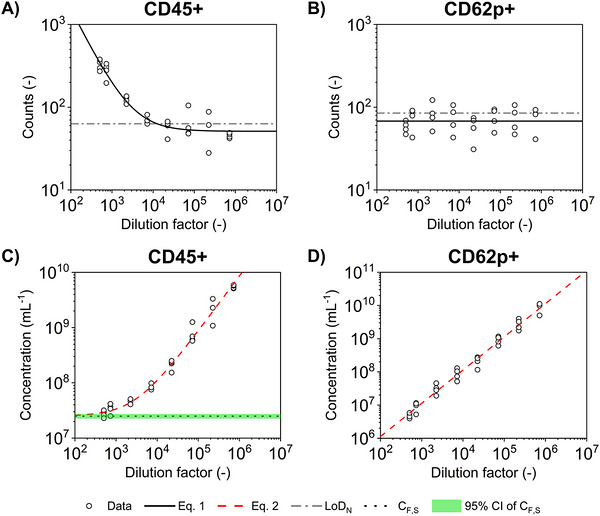
Spike‐in series of human very‐low‐density lipoprotein and chylomicrons into pooled plasma of 20 healthy volunteers in 4 replicates (Series 1). The total particle concentration increases with each spike‐in step, thereby also increasing the required dilution factor for scatter‐triggering measurements. All data represent events exceeding the side‐scatter threshold corresponding to a side‐scattering cross section of 3 nm^2^ with diameter < 1000 nm. (A) Measured number of events positive for CD45‐APC (> 36 MESF; symbols) versus the dilution factor fitted by Equation 1 (solid line). The dash‐dotted line represents the $LoD_{N}$ of 63 counts. Fit values: $N_{F,S}$ = 1.50 × 10^5^ ± 1.44 × 10^4^ counts; B = 5.16 × 10^1^ ± 3.15 counts. (B) Measured number of events positive for CD62p‐FITC (> 356 MESF; symbols) versus the dilution factor fitted by Equation 1 (solid line). The dash‐dotted line represents the $LoD_{N}$ of 85 counts. Fit values: $N_{F,S}$ = 0 ± 5.76 × 10^3^ counts; B = 6.80 × 10^1^ ± 4.88 counts. (C) Calculated concentration of CD45+ events versus the dilution factor (symbols). Fit values found in A were used in Equation 2 to evaluate the concentration versus dilution factor (dashed line). The dotted line with surrounding filled area represents the reference concentration of CD45+ events calculated using Equation 4 ($C_{F,S}$) ± standard error. $C_{F,S}$ = 2.49 × 10^7^ ± 2.40 × 10^6^ mL^−1^. (D) Calculated concentration of CD62p+ events versus the dilution factor (symbols). Fit values found in B were used in Equation 2 to evaluate the concentration versus dilution factor (dashed line). $C_{F,S}$= 0 ± 9.60× 10^5^ mL^−1^. APC = allophycocyanin; $C_{F,S}$, concentration of fluorescent events in the undiluted sample; CD, cluster of differentiation; CI, confidence interval; FITC, fluorescein isothiocyanate; $LoD_{N}$, limit of detection in terms of counts; MESF, molecules of equivalent soluble fluorochrome.

To characterize the correlation between the cTA+PC and the sample‐specific dilution factor, we prepared a series of plasma samples with increasing total particle concentration (Series 1 in Figure 3). Figure 4A and 4B show the dTA+PN (counts) versus dilution factor. The exact fit values are shown in Table S1. Ideally, a linear decrease in the dTA+PN is expected, leveling off at 0 counts. Figure 4A shows, however, that the CD45 dTA+PN levels off at 52 counts due to fluorescent background events. In Figure 4B no linear decrease in CD62p dTA+PN is observed, suggesting that all fluorescent events originate from the background. These results show that the number of background events differs between fluorescent markers and/or detectors.

Figure 4C and 4D show the cTA+PC, based on the dTA+PN in Figure 4A and 4B, versus the dilution factor. Ideally the cTA+PC is independent from the dilution factor and would follow the course illustrated by the green filled area in Figure 4C, which is the expected concentration obtained by solving Equation (2). However, the data shown in Figure 4C match the expected concentrations only for dilution factor < 10^3^, and at larger dilution factors the concentration is overestimated due to fluorescent background events. In Figure 4D no expected concentration could be determined because only background has been measured for CD62p. The data in Figure 4C and 4D show a linear increase in cTA+PC, illustrating the dependency of the concentration on the background events and dilution factor. Due to this dependency, concentrations of particles presumed to be labelled EVs measured at different dilution factors are incomparable.

### 4.2 Solution 1: Serial Dilution of Each Individual Sample

**FIGURE 5:**
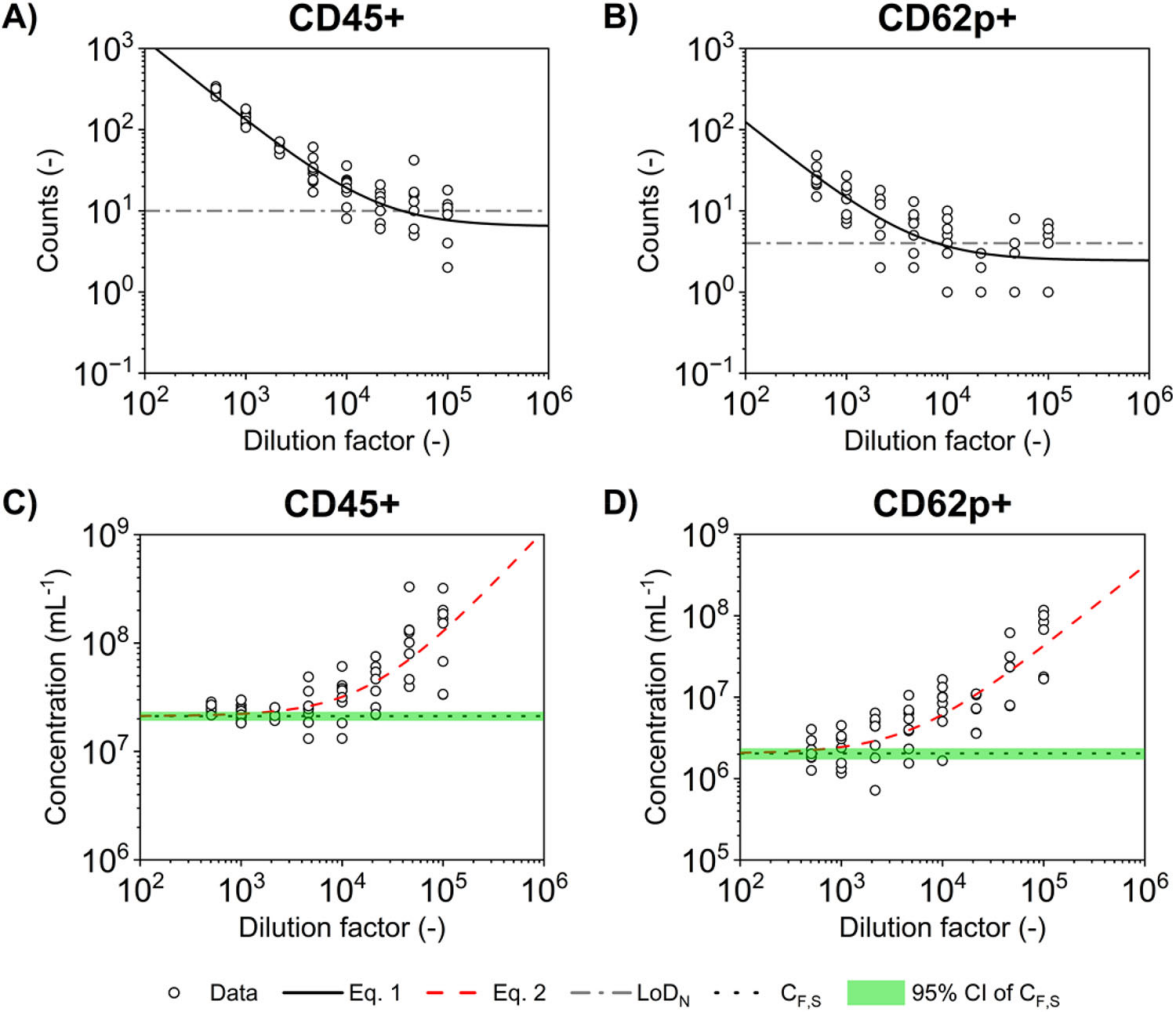
Serial dilution of pooled plasma of 20 healthy volunteers in 8 replicates (Series 2). All data represent events that exceeded the side‐scatter threshold corresponding to a side‐scattering cross section of 3 nm^2^ and with diameter <1000 nm. (A) Measured number of events positive for CD45‐APC (> 40 MESF; symbols) versus the dilution factor fitted by Equation (1) (solid line). The dashed line represents the $LoD_{N}$ of 10 counts. Fit values: $N_{F,S}$ = 1.27× 10^5^ ± 1.19 × 10^4^ counts; B = 6.42 ± 0.96 counts. (B) Measured number of events positive for CD62p‐FITC (> 450 MESF; symbols) versus the dilution factor fitted by Equation 1 (solid line). The dashed line represents the $LoD_{N}$ of 4 counts. Fit values: $N_{F,S}$ = 1.22 × 10^4^ ± 1.93 × 10^3^ counts; B = 2.45 ± 0.35 counts. (C) Calculated concentration of CD45+ events versus the dilution factor (symbols). Fit values found in A were used in Equation (2) to evaluate the concentration versus dilution factor (solid line). The dotted line with surrounding filled area represents the concentration of CD45+ events calculated using Equation (4) ($C_{F,S}$) ± standard error. $C_{F,S}$ = 2.12 × 10^7^ ± 1.98 × 10^6^ mL^−1^. (D) Calculated concentration of CD62p+ events versus the dilution factor (symbols). Fit values found in B were used in Equation (2) to evaluate the concentration versus dilution factor (solid line). The dotted line with surrounding filled area represents the concentration of CD62p+ events calculated using Equation (4) ($C_{F,S}$) ± standard error. $C_{F,S}$ = 2.04 × 10^6^ ± 3.22 × 10^5^ mL^−1^. APC, allophycocyanin; $C_{F,S}$, concentration of fluorescent events in the undiluted sample; CD, cluster of differentiation; CI, confidence interval; FITC, fluorescein isothiocyanate; $LoD_{N}$, limit of detection in terms of counts; MESF, molecules of equivalent soluble fluorochrome.

To obtain a comparable and reproducible value for the concentration of fluorescent particles in a sample, serial dilutions can be made of each sample. As an example, a serial dilution was made of a plasma sample in 8 replicates (Series 2, see Figure 3) and the results are shown in Figure 5. The exact fit values are also shown in Table S2. Figure 5A and 5B show the dTA+PN (counts) versus dilution factor, to which Equation (1) was fitted. The obtained fit values were used in Equation (4) to obtain the cTA+PC of the sample. In Figure 5C and 5D the $C_{F,S}$ for CD45+ event is 2.12 × 10^7^ ± 1.98 × 10^6^ mL^−1^ and the $C_{F,S}$ for CD62p+ events is 2.04 × 10^6^ ± 3.22 × 10^5^.

### 4.3 Solution 2: Use a Common Dilution Factor for All Samples

A lipoprotein spike‐in series (Series 3 in Figure 3) was made to show that a common dilution factor is another method to obtain comparable and reproducible concentrations of fluorescent events. Plasma was spiked with lipoproteins, thereby increasing the total particle concentration but leaving the EV concentration unaffected. Regardless of the spike‐in step, all samples were measured at a dilution factor of 500 using fluorescence‐triggering. For Series 3, scatter‐triggering was not possible, because samples with a total particle concentration >1·× 10^11^ mL^−1^ measured at a dilution factor of 500 cause swarm detection, which would bias the EV concentration measurement.

**FIGURE 6:**
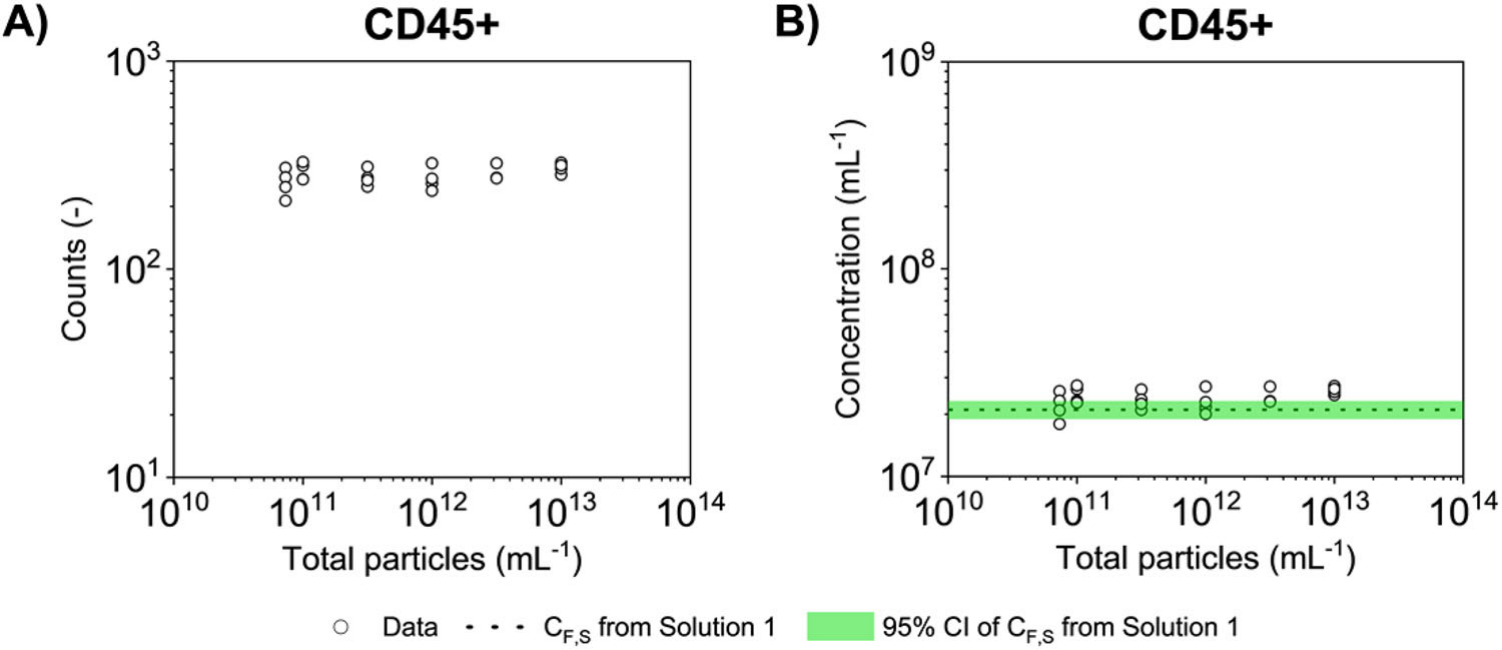
Spike‐in series of human very‐low‐density lipoprotein and chylomicrons in human pooled plasma of 20 healthy volunteers in 4 replicates (Series 3). The calculated concentration of CD45+ events (>60 MESF; symbols) remains stable with increasing total particle concentration when a constant dilution factor of 500 is used. Events were triggered on the APC detector at 29 arbitrary units corresponding to 32 APC‐MESF. (A) Number of measured CD45+ events versus total particle concentration. The total particle concentration was based on previous measurements and the spiked volume of lipoprotein sample. Counts based on all datapoints: 283 ± 32 with a coefficient of variation of 11.0%. (B) Calculated concentration of measured CD45+ events versus dilution factor. Concentration based on all datapoints: 2.39 × 10^7^ ± 2.64 × 10^6^ mL^−1^ with a coefficient of variation of 11.0%. The dotted line with surrounding filled area indicates CD45+ concentration ± standard error found using Solution 1 (Figure 5C). APC, allophycocyanin; CD, cluster of differentiation; CI, confidence interval; MESF, molecules of equivalent soluble fluorochrome.

Figure 6 shows that a common dilution factor and fluorescence triggering leads to a stable CD45 dTA+PN and therefore, a stable CD45 cTA+PC. Here, we excluded CD62p, because the number of CD62p particles is below the background for our test sample (Figure 4). The concentration of CD45+ events was 2.4 × 10^7^ ± 2.6 × 10^6^ mL^−1^. This concentration is similar to the concentration obtained by serial dilution using scatter‐triggering, see Figure S4. In addition, Figure S2A shows that a serial dilution measured using fluorescence‐triggering follows the expected curve of Equation (1), even at a dilution factor that would cause swarm detection by the scattering detector, that is, dilution factors <500 for this particular sample. This indicates that measurement without coincidence detection by the fluorescence detector would be feasible with fluorescence triggering. Thus, a common dilution factor and fluorescence triggering results in reliable and comparable concentration measurements of fluorescent events, regardless of the total particle concentration of a sample.

### 4.4 Retrospective Reliability Estimation May Change Clinical Study Outcomes

To retrospectively estimate the reliability of measurements from studies in which sample‐specific dilution was applied, we estimated $LoD_{N}$ and $DF_{max}$ (Equations 3 and 5) for each marker in two clinical studies that were performed in our laboratory previously (Buntsma et al., 2025; Gasecka et al., 2020).

**FIGURE 7:**
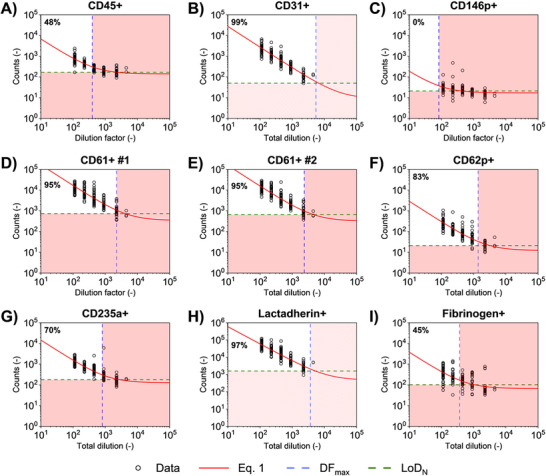
Estimation of assay performance in the clinical research study AFFECT EV (Gasecka et al., 2020) shows that not all data can be considered reliable. Data represent measured counts that exceeded the side scattering cross section threshold of 10 nm^2^, have a diameter < 1000 nm and are positive for the indicated marker versus dilution factor. The solid red line represents the fit of Equation (1) to the data. The vertical dashed line represents $DF_{max}$ and the horizontal dashed line represents $LoD_{N}$. All data within the shaded area are considered unreliable. The lighter shaded area in figures B and H indicate the uncertainty of the $LoD_{N}$ found for these markers, due to the insignificant *P*‐value of the fit of B. The upper left corner shows the estimated percentage of reliable datapoints. (A) CD45‐APC: $LoD_{N}$ = 169 counts, $DF_{max}$ = 3.94 × 10^2^, (B) CD31‐APC: $LoD_{N}$ = 50 counts, $DF_{max}$ = 5.46 × 10^3^, (C) CD146p‐PE: $LoD_{N}$ = 21 counts, $DF_{max}$ = 8.28 × 10^1^, (D) CD61‐APC #1: $LoD_{N}$ = 734 counts, $DF_{max}$ = 2.25 × 10^3^, (E) CD61‐APC #2: $LoD_{N}$ = 653 counts, $DF_{max}$ = 2.37 × 10^3^, (F) CD62p‐PE: $LoD_{N}$ = 21 counts, $DF_{max}$ = 1.38 × 10^3^, (G) CD235a‐PE: $LoD_{N}$ = 178 counts, $DF_{max}$ = 8.12 × 10^2^, (H) Lactadherin‐FITC: $LoD_{N}$ = 1608 counts, $DF_{max}$ = 3.68 × 10^3^, (I) Fibrinogen‐FITC: $LoD_{N}$ = 101 counts, $DF_{max}$ = 3.66 × 10^2^. Note that CD61‐APC was included in two separate duo‐stainings, therefore two measurements were made shown in figures D and E. APC, allophycocyanin; CD, cluster of differentiation; $DF_{max}$, maximum dilution factor; FITC, fluorescein isothiocyanate; $LoD_{N}$, limit of detection in terms of counts; PE, phycoerythrin.

In the AFFECT EV study, the $LoD_{N}$ ranged between 2.1 × 10^1^ and 1.6 × 10^3^ counts and $DF_{max}$ ranged between 8.2 × 10^1^ and 5.5 × 10^3^, see Figure 7. Based on the estimated limits shown in Figure 7 and Table S3, reliability of the study results is dependent on the label. For example, for CD146p (Figure 7C) none of the measurements (0%) is reliable based on our new insights, whereas for CD31 (Figure 7B) almost all (99%) of the measurements are reliable.

In the CINTICS study, the $LoD_{N}$ ranges between 1.1 × 10^1^ and 5.1 × 10^3^ counts and $DF_{max}$ ranged between 1.2 × 10^2^ and 1.8 × 10^4^. Based on the estimated limits shown in Figure S7 and Table S4, this study contained markers of which most measurements are reliable (such as CD31 with 93% reliable measurements), but the study also had markers with no reliable measurements (such as CD326 with 0% reliable measurements).

For none of the markers in both studies all measurements are reliable. This result shows how the dilution paradox can lead to overestimation of EV concentrations and thus misinterpretation of data.

## 5 Discussion

This study demonstrates that the concentration of EVs in samples calculated from flow cytometry measurements can be significantly overestimated due to the presence of fluorescent background events. The extent of overestimation increases with (i) the number of background events, and (ii) the total particle concentration and thus the dilution factor of a sample. Moreover, in the presence of fluorescent background events, concentrations of fluorescent particles in samples with different dilution factors cannot be compared to each other. Therefore, the outcomes of studies based on a sample‐specific dilution factor are unreliable, and sample‐specific dilution should not be used in EV flow cytometry experiments. Instead, a common dilution factor for all samples should be used.

To determine the concentration of fluorescently labelled EVs in plasma, the common dilution factor should be minimized, such that the number of fluorescent EVs in the measured sample volume is maximized. From the fluorescence perspective, a common dilution factor between 10 and 100‐fold is feasible for the flow cytometers used in this manuscript. From the light scattering perspective, however, a common dilution factor would need to exceed 1000‐fold to avoid swarm detection in samples with relatively high total particle concentration. We named this mismatch between the required dilution factor for fluorescence‐ and light scattering‐based detection of EVs the dilution paradox.

The dilution paradox was experimentally confirmed using dilution‐ and spike‐in series, and validated using published data from two independent clinical studies that were each carried out on different flow cytometers. Although we are confident that the concentration overestimations result from fluorescent background events combined with sample‐specific dilution, the source of the fluorescent background events itself remains unknown.

Aggregation of labelling reagents, like conjugated antibodies, protein aggregates or micelles may be a possible source of background events (Welsh et al., 2023). Formed protein aggregates can reach similar dimensions as EVs and emit a fluorescent signal, making them difficult to distinguish from labelled EVs. Even though centrifugation of labelling reagents reduces the number of aggregates, possibly not all aggregates are removed. A buffer with reagents control should always be used to confirm the absence of false‐positive counts resulting from aggregates of labelling reagents (Welsh et al., 2023).

Background events may also originate from non‐specific binding of the labelling reagents to non‐EV particles, such as lipoproteins. Although immunofluorescent staining is considered to be specific, antibodies can non‐specifically stick to non‐EV particles (Welsh et al., 2023). For increasing concentrations of non‐EV particles, the probability of non‐specific interactions between antibodies and non‐EV particles increases (Frutiger et al., 2021). Consequently, more background counts are expected in samples with a high total particle concentration than in samples with a low total particle concentration. This may explain the difference between background counts in our serial dilution (Figure 5) and lipoprotein spike‐in experiments (Figure 4).

Another possibility is that fluorescent background events originate from noise sources, such as fluidic noise and instrumental noise. To detect particles smaller than cells, such as EVs, flow cytometry signals might overlap with fluidic noise and are close to the LoD of the detectors (Bettin et al., 2023). Instrumental noise might therefore occasionally exceed the trigger threshold or gate. Although the origin of background events in EV flow cytometry falls outside the scope of this manuscript and requires more research, their impact on the measurement of particle concentrations is clear. This finding emphasizes the need for appropriate assay controls (Welsh et al., 2023), quantifying antibody titrations (Pink et al., 2023) and the removal of particulate contaminants from staining reagents.

Retrospective estimation of the reliability of results from the AFFECT EV and CINTICS studies confirm the effect of the dilution paradox (Buntsma et al., 2025; Gasecka et al., 2020). Neither study contained labels for which all measurements were reliable. Given the wide range of (un)reliability across labels, it is likely that study conclusions will change upon implementation of the estimated limits shown in the Supplementary Material. Based on this finding, study conclusions should be re‐evaluated.

It is important to emphasize that the retrospectively determined assay limits are estimates. These limits assume that the full study dataset would follow the shape of Equation (1). Equation (1) assumes that all fitted measurements share the same $N_{F,S}$, which is clearly not the case in datasets from clinical research studies. However, when Equation (1) was fitted to the study data of AFFECT EV and CINTICS, we found that in both studies the measured counts converged to a common background level with increasing sample‐specific dilution factor. This finding indicates that the dilution paradox can also be observed in the datasets from studies.

While the experimental evidence presented in this article is obtained using blood plasma, the dilution paradox may be relevant to other sample types as well. As an example, a recent study on the refractive index of EVs in human milk applied sample‐specific dilution factors between 300 and 15,000 (de Wolf et al., 2026). Moreover, cerebrospinal fluid has been reported to contain nanoparticle concentrations between 2 × 10^8^ and 7 × 10^9^ mL^−1^, also warranting sample‐specific dilutions (Welsh et al., 2024). The optimal pre‐analytical treatment of urine is still a topic of discussion, due to the dynamic composition of the samples resulting from fluid intake, collection time, medication, diet, exercise, age, biological sex and health status (Erdbrügger et al., 2021; Welsh et al., 2024). However, it has been shown that the total particle concentration of urine samples can differ at least one order of magnitude between donors, as also illustrated by a study in 2020 wherein the dilution factors applied to urine samples ranged between 10 and 100 (Kim et al., 2022, Rikkert et al., 2020). These examples illustrate that the dilution paradox should be considered when measuring both fluorescence and light scattering signals (e.g. for standardization purposes) of EVs in blood plasma and other sample types in which particle concentrations differ by orders of magnitude between samples.

Regardless of sample type, it is no surprise that reliable concentration measurements of EVs by flow cytometry is challenging. Reported EV concentrations are the product of instrument characteristics (such as detector sensitivity) and experimental parameters (such as flow rate and dilution). In recent years, efforts have been made to standardize EV concentration measurements through calibration and standardized reporting (Bettin et al., 2023; Welsh et al., 2020). Although comparability across instruments and institutes has improved (Bettin et al., 2025), our present findings show that the method we used and recommended to prevent swarm detection by light scattering actually leads to incomparable concentration measurements of fluorescently labelled EVs. This raises the question of how future EV flow cytometry assays should be set up to obtain reliable concentration measurements.

Many parameters must be considered when setting up an EV flow cytometry assay (Welsh et al., 2023). Here we will focus on how to handle the dilution paradox. At present, there is no consensus on the optimal triggering strategy for EV detection (Arraud et al., 2016; Nolan and Duggan, 2018; Stoner et al., 2016; Welsh et al., 2017). In our present manuscript, we show that in studies such as AFFECT EV or CINTICS, where the total particle concentration varies ≥2 orders of magnitude, scatter‐triggering does not result in reliable EV concentration measurements for all samples due to the need for sample‐specific dilution. In theory, scatter‐triggering can be applied when the variation in total particle concentration between samples is limited. However, the total particle concentration variation between samples is usually unknown before the start of the study. Fluorescence‐triggering allows the use of a common dilution factor for all samples, which results in measuring a comparable concentration of EVs that is independent from the sample dilution. Therefore, triggering on fluorescence is a safe choice regardless of the variation in total particle concentration across samples. However, using fluorescence‐triggering with a common dilution factor can lead to swarm detection on the scatter detector for samples with a relatively high total particle concentration, as was described previously (Libregts et al., 2018). This impairs scatter‐based size calibration of these samples (van der Pol et al., 2018).

Size calibration cannot be ignored, because it provides essential information that is needed to distinguish between, for example, EVs and platelets (Bettin et al., 2023). In addition, size calibration is a proven way to achieve comparable EV concentration measurements between different flow cytometers (van der Pol et al., 2018). Although serial dilutions enable comparable and reproducible concentration measurements using scatter‐triggering, at the same time such dilutions are laborious, time‐consuming, and expensive.

Alternatively, fluorescently stained liposomal standards can be used for fluorescence‐based size calibration (Sandau et al., 2020; Stoner et al., 2016). In this approach, the fluorescence intensity distribution of a well‐characterized liposome population is related to the surface area distribution of EVs labelled with a lipid dye to approximate the EV size distribution. However, lipid dyes may exhibit broad emission spectra, leading to spectral spillover that reduces sensitivity in other detection channels. Furthermore, whereas scattering‐based calibration relies on the accuracy of the assumed EV refractive index (de Rond et al., 2018), fluorescence‐based size calibration relies on the assumption that EVs and liposomal standards are labelled with a comparable fluorophore density. Given the heterogeneous nature of EVs, both assumptions are likely to hold only on average at the population level. A new interlaboratory study may therefore be valuable to evaluate and refine these underlying assumptions, although such efforts are beyond the scope of this article.

Next to size calibration, sample processing is also an important topic in EV flow cytometry. Even when fluorescence‐triggering with a common dilution factor is used, all measurements will contain fluorescent background counts. However, with a common dilution factor, the background contribution to the concentration measurement of labelled EVs becomes independent of the dilution factor. Still, it is good practice to maximize the signal‐to‐background ratio by considering the use of different sample processing techniques (such as size‐exclusion‐chromatography) and optimized reagent processing techniques (such as antibody titration and centrifugation). Controlling the fasting status of the donors may also mitigate background events. Generally, identifying possible sources of background in each specific situation and anticipating accordingly can improve the quality of measurements.

Fluorescence‐based triggering of EVs is not straightforward, as the ability to distinguish EVs from the background depends on multiple factors, including label affinity, the number of binding sites, fluorophore brightness, and detector sensitivity. To minimize the dependence of an assay on label affinity and binding‐site availability, EVs are typically labelled with an excess of reagents to drive the reaction towards saturation. However, this excess may require removal of reagents through additional sample processing steps. In addition, differences in detector sensitivities can result in one label being detectable while another is not. For example, the instrument used in this study has a detection efficiency of 0.06 photoelectrons per MESF for APC and 0.005 photoelectrons per MESF for FITC (MIFlowCyt‐EV section 3.2.3). The lower sensitivity for FITC might partly explain why all CD62p+ events overlap with background events in Figure 4. Regardless of the fluorophore used, CD62p+ EVs in plasma are generally less abundant than CD45+ EVs, making them more challenging to detect statistically. Together, these considerations highlight the complexity of factors influencing the quality and interpretation of EV measurements by flow cytometry.

To get an indication of the quality of measurements, we recommend performing a serial dilution of at least one sample per study group per marker. Assessing this serial dilution may give an indication whether the measurement contains just background, or a signal from labelled EVs as well. When this control confirms that the measurement does not consist of only background events, the remaining samples can be measured at a common dilution factor using fluorescence triggering. As the common dilution factor will depend on the flow cytometer and sample type, it will require expertise to select the optimal dilution factor. The optimal dilution factor will be a compromise between background (dilution factor too high) and swarm detection (dilution factor too low).

A summary of the methodological recommendations given in this article can be found in Table 1. Currently, we are investigating how to standardize the selection of the dilution factor and the optimal fluorescence trigger threshold. In addition, we are investigating if scatter‐based size calibration could be compatible with fluorescence‐triggering and whether exclusion criteria, such as a maximum total particle concentration, might be required in clinical studies on plasma. Further work is needed to validate such findings across different flow cytometers.

**TABLE 1:** Methodological recommendations towards reliable extracellular vesicle (EV) concentration measurements using flow cytometry. EV, extracellular vesicle.

Problem	Recommendation	Advantages	Disadvantages
Occurrence of swarm detection when using scatter‐triggering to measure EVs in samples with relatively high total particle concentration	Use fluorescence‐triggering	Allows for the use of a common dilution factor for all samples, including samples with high total particle concentration	Occurrence of swarm detection on the scatter detector limits the amount of information that can be reliably obtained from the instrument.
Sample‐specific dilution factors lead to incomparable EV concentrations	Use the same dilution factor for all samples in the same study	EV concentrations are directly comparable to each other	Choosing a common dilution factor is challenging due to the dilution paradox
Total particle concentration of samples differs between different donors, such that sample‐specific dilution factors would be applied when scatter‐triggering is used	Apply donor inclusion criteria based on fasting status, water intake, collection time, etc.	Limits the total particle concentration in the sample	Not possible in studies where sample collection is performed in acute conditions
	Apply exclusion criteria for samples based on total particle concentration, that is, a maximum total particle concentration	All included samples are ensured to be measured within the dynamic range of the assay	More samples required to account for exclusion of part of the samples
	Use fluorescence triggering	See above	See above
Background events bias EV concentration measurements	Identify possible sources of background events, such as residual platelets, antibody aggregates, non‐specific labelling or instrument noise	Awareness of the possible sources of background events facilitates more straightforward interpretation of the data	
Background events originate from the sample	Use EV isolation techniques, such as size exclusion chromatography or ultrafiltration	Reduces the number of irrelevant particles in the measurement, which may reduce the number of background events	Expensive, time consuming, and EV recovery may not reach 100%
Background events originate from antibody aggregates	Centrifuge antibody solution and add only the supernatant to the sample	Easy, fast, and effective	Few percent loss of antibodies
Background events remain present despite mitigation measures	Apply assay controls to confirm that signals originate from labelled EVs	Improves reliability, reproducibility and comparability	Effect of detergents on non‐EV particles in the sample is not well‐characterized
Unclear whether low concentration measurements contain only background	Make a serial dilution of one sample per study group per marker	Gives an indication whether the measurements contain relevant EV concentrations or just background for that particular marker and study group	Laborious, time consuming and requires more reagents than single measurements.

## 6 Conclusion

Fluorescent background events in combination with sample‐specific dilution leads to overestimated and incomparable concentration measurements between samples due to a phenomenon that we named the ‘dilution paradox’. Consequently, results and conclusions from studies that applied sample‐specific dilution may not be reliable and thus, should be reconsidered. Comparable and reproducible concentration measurements of samples that differ in total particle concentration can be obtained by (i) using scatter‐triggering to measure a serial dilution of each sample (not recommended), or (ii) using fluorescence‐triggering to measure all samples at identical dilution factors (recommended), thereby risking swarm detection by the light scattering detector for a fraction of the samples. The methodological recommendations given here will contribute to more reproducible and comparable EV concentration measurements and, ultimately, to accelerated clinical implementation of EV flow cytometry

## Author Contributions

**Joyce Rops**: conceptualization, data curation, formal analysis, methodology, project administration, visualization, writing – original draft, writing – review and editing, investigation, validation. **Naomi Buntsma**: data curation, investigation, resources, writing – review and editing. **Aleksandra Gąsecka**: data curation, investigation, resources, writing – review and editing. **Leonie de Rond**: data curation, formal analysis. **Nyika D. Kruyt**: funding acquisition, supervision, writing – review and editing. **Rienk Nieuwland**: conceptualization, methodology, resources, supervision, writing – original draft, writing – review and editing. **Edwin van der Pol**: conceptualization, funding acquisition, methodology, resources, supervision, writing – original draft, writing – review and editing, software, formal analysis.

## Funding

Joyce Rops acknowledges funding from the Dutch Heart Foundation, Impulse Grant CINTICS II (01‐002‐2023‐0142). Edwin van der Pol acknowledges funding from the Dutch Research Council (NWO), VIDI grant 19724.

## Conflicts of Interest

Edwin van der Pol is co‐founder and shareholder of Exometry (Amsterdam, the Netherlands). All other authors have no relevant conflicts of interest to declare.

## Supporting information




**Supporting Information**: jev270312‐sup‐0001‐SuppMat.docx

**Supporting Information**: jev270312‐sup‐0002‐SuppMat.docx

## Data Availability

Data generated in this study are available upon request from the corresponding author. The data that support the findings of this study are openly available in Figshare at https://figshare.com/s/9102e9cc0ce8a0bb5ede, reference number 10.6084/m9.figshare.31073530.
