# Supplementary material for: The Dilution Paradox in Extracellular Vesicle Flow Cytometry
Source: J Extracell Vesicles. 2026 Jun 23;15(6):e70312. doi: 10.1002/jev2.70312 (PMC13291209; doi:10.1002/jev2.70312)
Supplement: Supplementary file 1 — Supporting Information: jev270312‐sup‐0001‐SuppMat.docx [file JEV2-15-e70312-s002.docx]

**The dilution paradox in extracellular vesicle flow cytometry**

Supplementary material

**
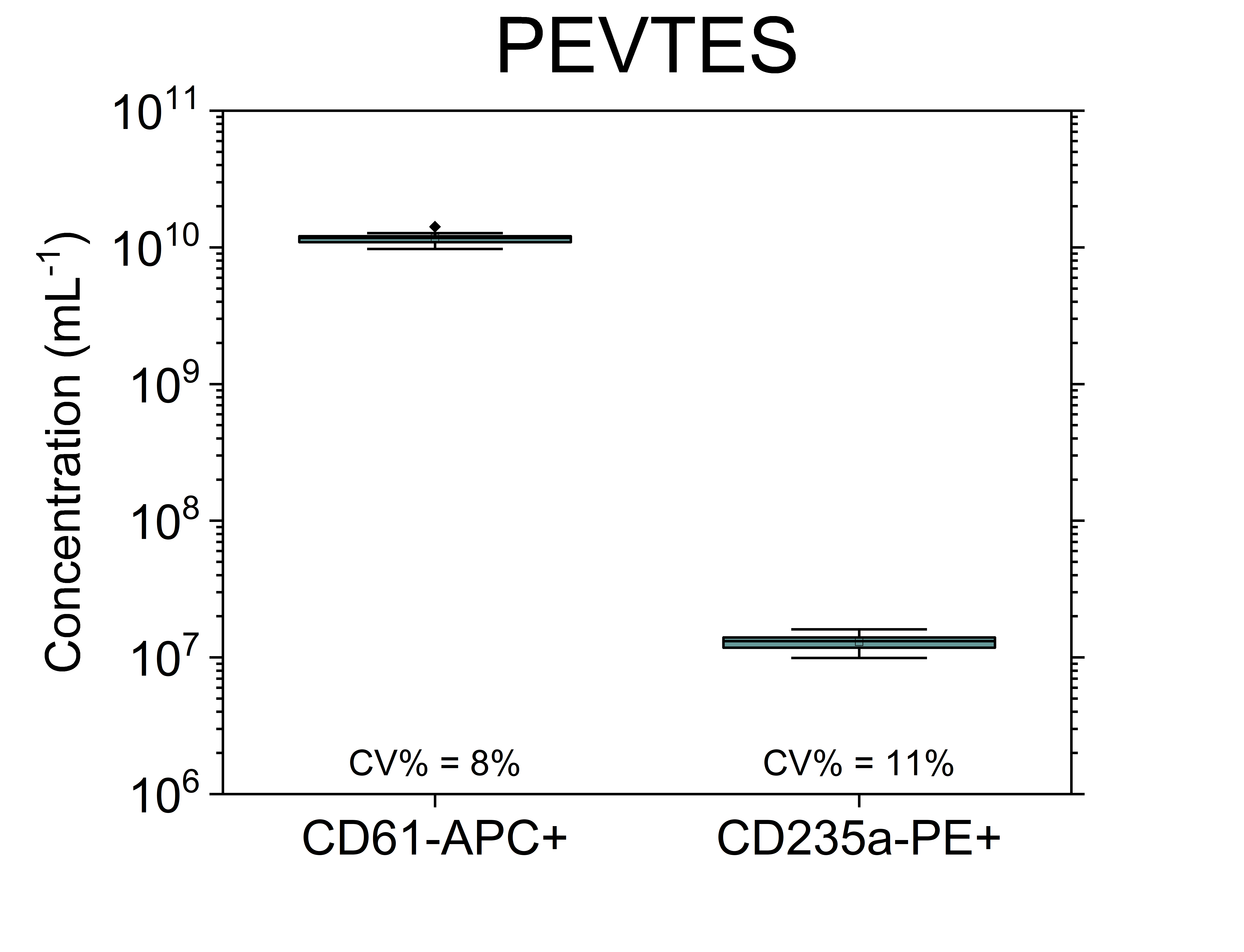
Measurement precision**

**Supplementary Figure 1:** Concentration measured in 20 plasma EV test samples on 20 different days by 7 different operators. Boxplot of measured concentrations of particles that exceeded the side scatter cross section threshold of 3 nm^2^, have a diameter < 1,000 nm and are positive for (left) CD61-APC or (right) CD235a-PE. The measured concentration of CD61-APC positive particles is 1.17·10^10^ ± 9.88·10^8^ mL^-1^ with a coefficient of variation of 8%. The measured concentration of CD235a-PE positive particles is 1.30·10^7^ ± 1.47·10^6^ mL^-1^ with a coefficient of variation of 11%. APC = allophycocyanin; CD = cluster of differentiation; MESF = molecules of equivalent soluble fluorochrome; PE = phycoerythrin; PEVTES = Plasma EV Test Sample.

Concentration precision of our flow cytometer settings (Apogee A60-Micro, Apogee Flow Systems) was confirmed using 20 plasma EV test sample (PEVTES) measurements from 20 different days. PEVTES is a pre-stained ready-to-use test sample that resembles subcellular particles in plasma (1). PEVTES used in this article was made by collecting EVs from a platelet concentrate. The samples were double stained with CD61-APC (cat. 17-0619-42, lot. 2062626, VI-PL2, Invitrogen, final concentration 8.33 µg/mL) and CD235a-PE (cat. R7078, lot. 41236187, JC159, Dako Agilent, final concentration 25 µg/mL). A buffer of trehalose-dihydrate (cat. 625625-50GM, lot. 4104554, Millipore, final concentration 0.5 M) was added to stabilize the sample. After that 5% bovine serum albumin was added to the samples (cat. A9647-100G, lot. SLBK0462V, Sigma Aldrich, final concentration 0.5% w/v). The stability of the EV concentration in PEVTES has been shown before (2).

Supplementary Figure 1 shows the PEVTES concentration measurements and their coefficient of variation (CV). The CVs of both measurements indicates that with the settings used our flow cytometer is capable of precise and repeatable concentration measurements, independent of the operator or measurement day.

**Absence of coincidence detection with fluorescence triggering**


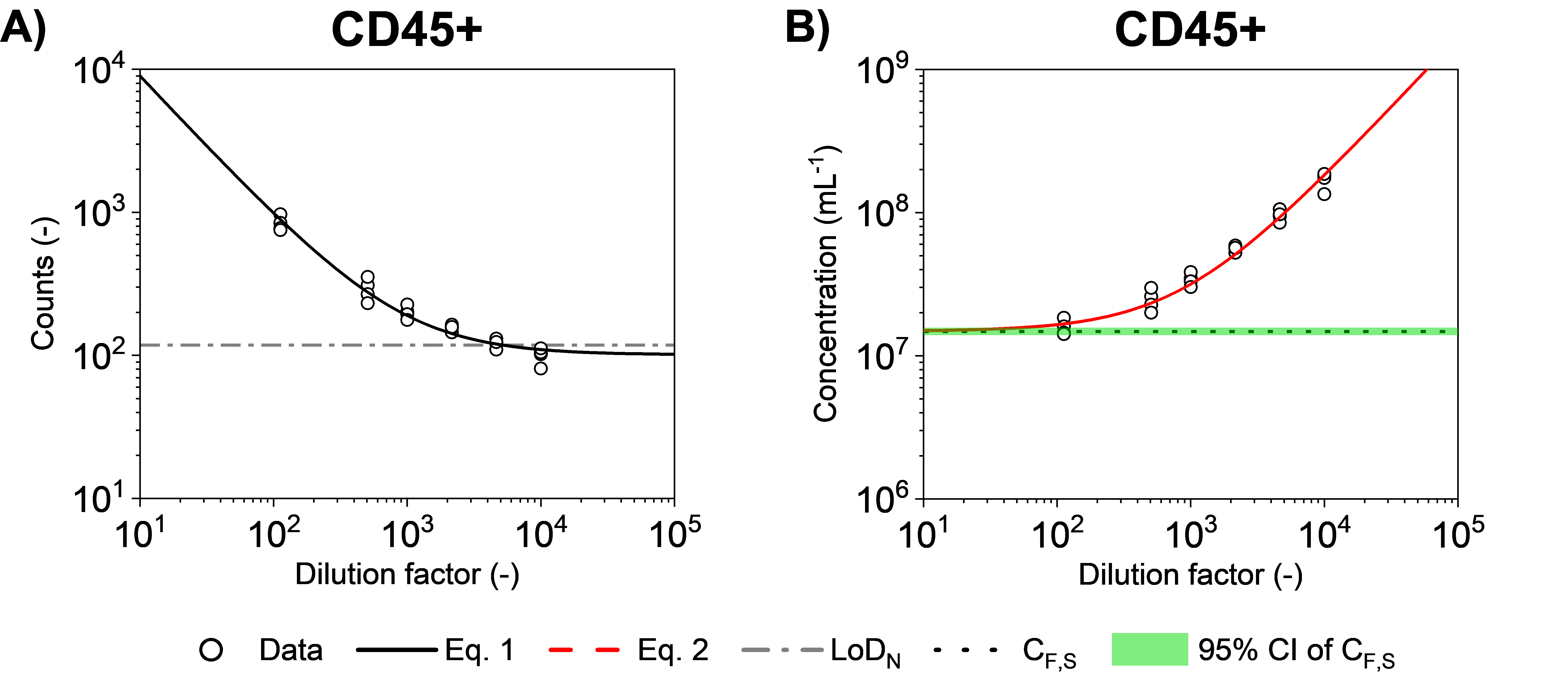


**Supplementary Figure 2**: Serial dilution of pooled plasma of 20 healthy volunteers in 4 replicates. All events were triggered on the APC detector at 29 arbitrary units corresponding to 32 APC-MESF. (A) Measured number of events positive for CD45-APC (> 60 MESF; symbols) versus the dilution factor fitted by Equation 1 (solid line). The dashed line represents the ${LoD}_{N}$ of 117 counts. Fit values: $N_{F,S}$ = 8.89·10^4^ ± 5.38·10^3^ counts; $B$ = 1.01·10^2^ ± 4.80 counts. (B) Calculated concentration of CD45+ events versus the dilution factor (symbols). Fit values found in A were used in Equation 2 to evaluate the concentration versus dilution factor (solid line). The dotted line with surrounding filled area represents the concentration of CD45+ events calculated using Equation 4 ($C_{F,S}$) ± standard error. $C_{F,S}$ = 1.48·10^7^ ± 8.97·10^5^ mL^-1^. APC = allophycocyanin; $C_{F,S}$ = concentration of fluorescent events in the undiluted sample; CD = cluster of differentiation; CI = confidence interval; ${LoD}_{N}$ = limit of detection in terms of counts; MESF = molecules of equivalent soluble fluorochrome.

In order to confirm the absence of coincidence detection when using fluorescence triggering, serial dilutions were made of human pooled plasma labelled with CD45-APC. For this experiment dilution factors ranging from 1·10^2^ to 1·10^4^ were chosen, as swarm detection would be expected with scatter triggering detection at the lower end of this range. Particles were detected using flow cytometry with a fluorescence trigger threshold of 29 arbitrary units, corresponding to 32 APC-MESF.

Supplementary Figure 2 shows that even at the lowest dilution factor of the serial dilution no swarm detection by the fluorescent detector was observed, because the events follow equation 1. As the lowest dilution factor used in Supplementary Figure 2 was expected to cause swarm detection by the scatter channel, this result indicates that with fluorescence triggering a common low dilution factor for all samples would be feasible.

**Swarm detection on the scatter channel does not affect concentration measurements using fluorescence triggering**

**
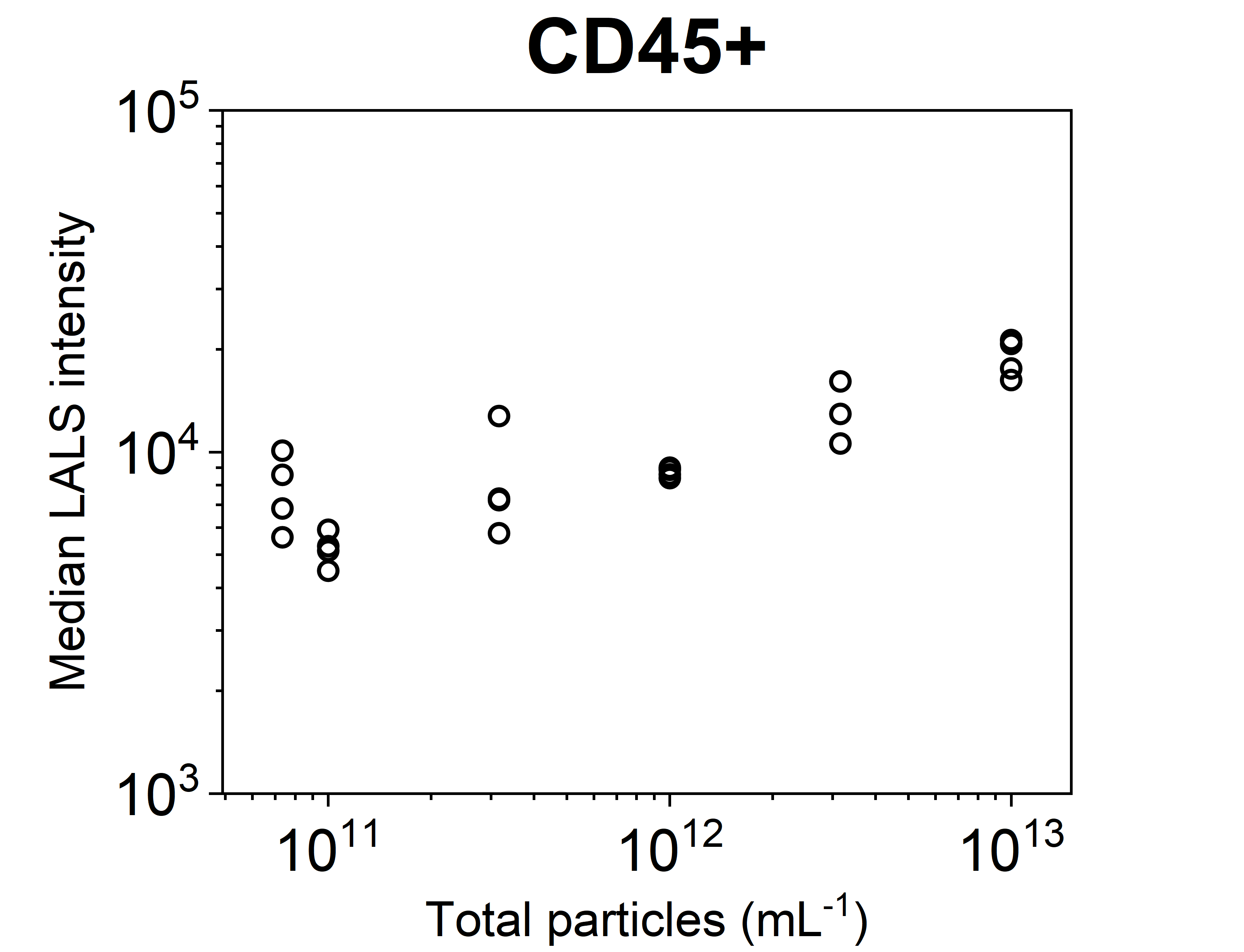
**

**Supplementary Figure 3:** Median LALS signal intensity increases with the total particle concentration, indicating that swarm detection starts to increase on the LALS channel with increasing total particle concentration. Events were triggered on the APC detector at 29 arbitrary units corresponding to 32 APC-MESF, and positive for CD45 (>60 APC-MESF). APC = allophycocyanin; CD = cluster of differentiation; LALS = large angle light scattering; MESF = molecules of equivalent soluble fluorochrome.

The increase of the median LALS intensity with total particle concentration shown in Supplementary Figure 3 indicates that swam detection occurred increasingly on the scatter channel. These results show that even though swarm detection is present on the scatter channel, the CD45+ concentration measured with fluorescence-triggering remained constant with increasing total particle concentration. We attribute the data spread to the limited number of CD45+ events together with the heterogeneous nature of EVs.

**Both triggering strategies can yield comparable concentration measurements**

In this study we have shown two strategies to obtain a reliable EV concentration measurement: one with scatter-triggering and one with fluorescence triggering. Leukocyte-derived EV concentrations have been measured using both strategies. Supplementary Figure 3 shows the comparison between concentrations measured at the same dilution factor of 500 with scatter- or fluorescence-triggering. Statistical testing using one-way ANOVA with confidence level 0.05 showed no significant difference between the concentrations.


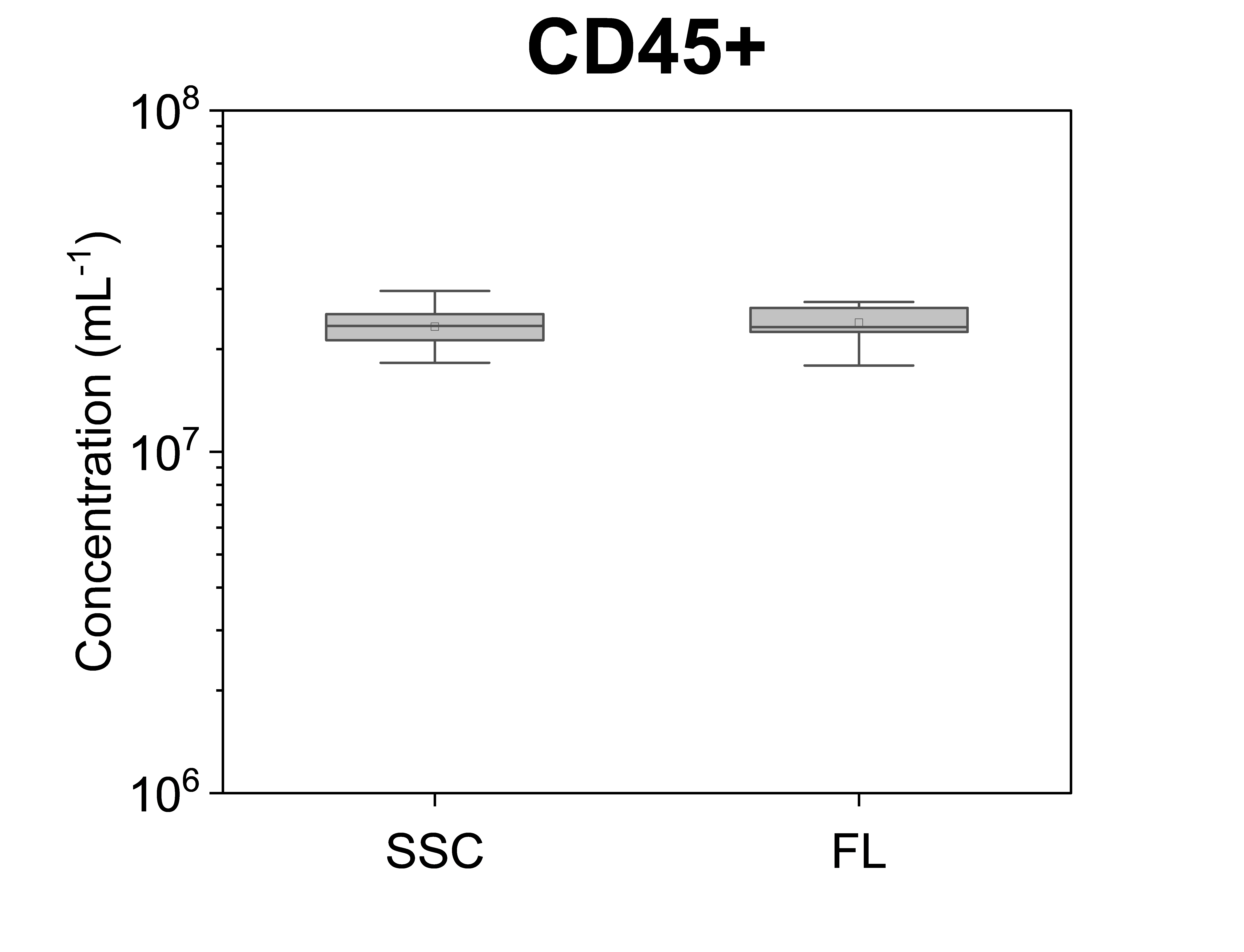


**Supplementary Figure 4:** Comparing extracellular vesicle concentrations using scatter-triggering (SSC) and fluorescence-triggering (FL) shows no significant difference between the measurement strategies. (SSC) All data represents particles with diameter < 1,000 nm exceeding the side scatter threshold, which corresponds to a side scatter cross section of 3 nm^2^. (FL) All data represent particles exceeding the fluorescence threshold of 29 arbitrary units, corresponding to 32 APC-MESF. APC = allophycocyanin; MESF = molecules of equivalent soluble fluorochrome.



**Supplementary Figure 7**: Estimation of assay performance in the clinical study CINTICS shows that not all data can be considered reliable. Data represent measured counts that exceeded the side scatter cross section threshold of 2 nm^2^, have a diameter between 100 and 1,000 nm and are positive for the indicated marker versus dilution factor. The solid red line represents the fit of Equation 1 to the data. The vertical dashed line represents $DF_{max}$ and the horizontal dashed line represents $LoD_{N}$. All data within the shaded area are considered unreliable. The upper left corner shows the estimated percentage of reliable datapoints. (A) GSAO-AF647: $LoD_{N}$ = 23 counts, $DF_{max}$ = 1.49·10^3^, (B) CD45-APC: $LoD_{N}$ = 127 counts, $DF_{max}$ = 6.79·10^3^, (C) CD235a-PE: $LoD_{N}$ = 201 counts, $DF_{max}$ = 8.41·10^3^, (D) CD14-PacificBlue: $LoD_{N}$ = 67 counts, $DF_{max}$ = 7.88·10^2^, (E) CD326-APC: $LoD_{N}$ = 82 counts, $DF_{max}$ = 1.28·10^2^, (F) CD62p-PE: $LoD_{N}$ = 10 counts, $DF_{max}$ = 1.58·10^3^, (G) CD41-PacificBlue: $LoD_{N}$ = 150 counts, $DF_{max}$ = 6.59·10^3^, (H) CD31-APC: $LoD_{N}$ = 30 counts, $DF_{max}$ = 2.43·10^4^, (I) CD146-PE: $LoD_{N}$ = 45 counts, $DF_{max}$ = 1.74·10^2^. APC = allophycocyanin; CD: cluster of differentiation; $DF_{max}$ = maximum dilution factor; $LoD_{N}$ = limit of detection in terms of counts; PE = phycoerythrin.

**Supplementary table 1:** Values obtained after fitting Equation 1 to log_10_-transformed data of a lipoprotein spike-in series measured with scatter-triggering (Figure 3 in main article). Fit values $N_{F,S}$ and $B$ are represented as mean ± standard error. The reported P-value is the probability that the null hypothesis of the t-test that the fit value equals 0 is true. P < 0.05 is considered significant.

| Marker | N_F,S_ | P-value | B | P-value | LoD_N_ | C_F,S_ |
| --- | --- | --- | --- | --- | --- | --- |
| CD45 | 1.50·10^5^±1.44·10^4^ | <0.0001 | 5.16·10^1^±3.15 | <0.0001 | 63 | 2.49·10^7^±2.40·10^6^ |
| CD62p | 0 ± 5.76·10^3^ | 1.0000 | 6.80·10^1^ ± 4.88 | <0.0001 | 85 | 0±9.60·10^5^ |

**Supplementary table 2:** Values obtained after logistic regression fitting Equation 1 to log_10_-transformed data of a serial dilution series measured with scatter-triggering (Figure 4 in main article). Fit values $N_{F,S}$ and $B$ are represented as mean ± standard error. The reported P-value is the probability that the null hypothesis of the t-test that the fit value equals 0 is true. P < 0.05 is considered significant.

| Marker | N_F,S_ | P-value | B | P-value | LoD_N_ | C_F,S_ |
| --- | --- | --- | --- | --- | --- | --- |
| CD45 | 1.27·10^5^±1.19·10^4^ | <0.0001 | 6.42±9.57·10^-1^ | <0.0001 | 10 | 2.12·10^7^±1.98·10^6^ |
| CD62p | 1.22·10^4^±1.93·10^3^ | <0.0001 | 2.45±3.50·10^-1^ | <0.0001 | 4 | 2.04·10^6^±3.22·10^5^ |

**Supplementary table 3:** Estimated assay performance found after fitting Equation 1 to the data of the clinical study AFFECT EV (Figure 6 in main article). Fit values $N_{F,S}$ and $B$ are represented as mean ± standard error. The reported P-value is the probability that the null hypothesis of the t-test that the fit value equals 0 is true. P < 0.05 is considered significant. % reliable indicates the percentage of datapoints that could be considered reliable based on the estimated assay limits.

| Marker | N_F,S_ | P-value | B | P-value | LoD_N_ | DF_max_ | % reliable |
| --- | --- | --- | --- | --- | --- | --- | --- |
| CD45 | 6.64·10^4^±3.52·10^3^ | <0.0001 | 1.40·10^2^±8.82 | <0.0001 | 169 | 3.94·10^2^ | 48 |
| CD31 | 2.73·10^5^±1.24·10^4^ | <0.0001 | 8.95±1.24·10^1^ | 0.4727 | 50 | 5.46·10^3^ | 99 |
| CD146p | 1.70·10^3^±2.76·10^2^ | <0.0001 | 1.70·10^1^±1.07 | <0.0001 | 21 | 8.28·10^1^ | 0 |
| CD61 #1 | 1.65·10^6^±9.79·10^4^ | <0.0001 | 3.42·10^2^±1.19·10^2^ | 0.0044 | 734 | 2.25·10^3^ | 95 |
| CD61 #2 | 1.54·10^6^±8.41·10^4^ | <0.0001 | 3.20·10^2^±1.01·10^2^ | 0.0017 | 653 | 2.37·10^3^ | 95 |
| CD62p | 2.87·10^4^±1.77·10^3^ | <0.0001 | 1.22·10^1^±2.57 | <0.0001 | 21 | 1.38·10^3^ | 83 |
| CD235a | 1.44·10^5^±8.28·10^3^ | <0.0001 | 1.27·10^2^±1.53·10^1^ | <0.0001 | 178 | 8.12·10^2^ | 70 |
| Lactadherin | 5.91·10^6^±3.21·10^5^ | <0.0001 | 4.77·10^2^±3.42·10^2^ | 0.1653 | 1608 | 3.68·10^3^ | 97 |
| Fibrinogen | 3.70·10^4^±4.37·10^3^ | <0.0001 | 6.71·10^1^±1.03·10^1^ | <0.0001 | 101 | 3.66·10^2^ | 45 |

**Supplementary table 4:** Estimated assay performance found after fitting Equation 1 to the data of the clinical study CINTICS EV-FCM. Fit values N_F,S_ and B are represented as mean ± standard error. The reported P-value is the probability that the null hypothesis of the t-test that the fit value equals 0 is true. P < 0.05 is considered significant. % reliable indicates the percentage of datapoints that could be considered reliable based on the estimated limits.

| Marker | N_F,S_ | P-value | B | P-value | LoD_N_ | DF_max_ | % reliable |
| --- | --- | --- | --- | --- | --- | --- | --- |
| CD45 | 8.66·10^5^±6.11·10^4^ | <0.0001 | 7.80·10^1^±1.50·10^1^ | <0.0001 | 127 | 6.79·10^3^ | 81 |
| CD31 | 7.26·10^5^±4.19·10^4^ | <0.0001 | 9.75±6.10 | 0.112 | 30 | 2.43·10^4^ | 96 |
| CD146p | 7.86·10^3^±4.25·10^3^ | 0.0662 | 3.46·10^1^±3.21 | <0.0001 | 45 | 1.74·10^2^ | 0 |
| CD41 | 9.87·10^5^±9.57·10^4^ | <0.0001 | 7.56·10^1^±2.25·10^1^ | 9.72·10^-4^ | 150 | 6.59·10^3^ | 75 |
| CD62p | 1.64·10^4^±2.54·10^3^ | <0.0001 | 6.97±1.05 | <0.0001 | 10 | 1.58·10^3^ | 31 |
| CD235a | 1.69±10^6^±1.10·10^5^ | <0.0001 | 1.18·10^2^±2.50·10^1^ | <0.0001 | 201 | 8.41·10^3^ | 84 |
| CD14 | 5.28·10^4^±9.15·10^3^ | <0.0001 | 5.09·10^1^±4.87 | <0.0001 | 67 | 7.88·10^2^ | 13 |
| CD326 | 1.05·10^4^±5.21·10^3^ | 0.0456 | 6.74·10^1^±4.34 | <0.0001 | 82 | 1.29·10^2^ | 0 |
| GSAO | 3.44·10^4^±7.93·10^3^ | <0.0001 | 1.28·10^1^±3.13 | <0.0001 | 23 | 1.49·10^3^ | 25 |
