# Supplementary material for: The Dilution Paradox in Extracellular Vesicle Flow Cytometry
Source: J Extracell Vesicles. 2026 Jun 23;15(6):e70312. doi: 10.1002/jev2.70312 (PMC13291209; doi:10.1002/jev2.70312)
Supplement: Supplementary file 2 — Supporting Information: jev270312‐sup‐0002‐SuppMat.docx [file JEV2-15-e70312-s001.docx]

MIFlowCyt-EV of study “The dilution paradox in extracellular vesicle flow cytometry”

This document aims to provide the minimum information required to reproduce the flow cytometry experiments in the study “The dilution paradox in extracellular vesicle flow cytometry”. This document is based on three published standardization frameworks and guidelines [1–3].

**Contents**

[1 Experiment overview 2](#_Toc222819258)

[1.1 Contact details 2](#_Toc222819259)

[1.1.1 Experiment leader 2](#_Toc222819260)

[1.1.2 Flow cytometry execution 2](#_Toc222819261)

[1.2 Purpose 2](#_Toc222819262)

[1.3 Keywords 2](#_Toc222819263)

[1.4 Experiment variables 2](#_Toc222819264)

[1.5 Experiment design and quality controls 3](#_Toc222819265)

[1.6 Dates 3](#_Toc222819266)

[1.7 Conclusions 3](#_Toc222819267)

[2 Sample details 3](#_Toc222819268)

[2.1 Sample description 3](#_Toc222819269)

[2.1.1 Sample source description 3](#_Toc222819270)

[2.1.2 Sample description 3](#_Toc222819271)

[2.2 Sample collection 4](#_Toc222819272)

[2.3 Sample storage 4](#_Toc222819273)

[2.4 Sample characteristics 4](#_Toc222819274)

[2.5 Sample staining 4](#_Toc222819275)

[2.6 Fluorescence reagents 6](#_Toc222819276)

[3 Flow cytometer 8](#_Toc222819277)

[3.1 Model and manufacturer 8](#_Toc222819278)

[3.2 Configuration and settings 8](#_Toc222819279)

[3.2.1 Flow rate and acquisition time 8](#_Toc222819280)

[3.2.2 Light sources 8](#_Toc222819281)

[3.2.3 Detectors 8](#_Toc222819282)

[3.2.4 Trigger detector and threshold 8](#_Toc222819283)

[4 Assay controls 9](#_Toc222819284)

[4.1 Unstained controls 9](#_Toc222819285)

[4.2 Reagent-in-buffer controls 10](#_Toc222819298)

[4.3 Detergent lysis controls 11](#_Toc222819299)

[4.4 Buffer only controls 13](#_Toc222819301)

[4.5 Isotype controls 14](#_Toc222819302)

[5 Data analysis 16](#_Toc222819303)

[5.1 Data sharing 16](#_Toc222819304)

[5.2 Compensation details 16](#_Toc222819305)

[5.3 Calibrations 16](#_Toc222819306)

[5.3.1 Flow rate 16](#_Toc222819307)

[5.3.2 Fluorescence calibration 16](#_Toc222819308)

[5.3.3 Light scattering calibration 18](#_Toc222819310)

[5.4 Gate description and boundaries 19](#_Toc222819311)

[6 References 22](#_Toc222819312)

# Experiment overview

## Contact details

### Experiment leader

| Name of organization | Laboratory of Experimental Clinical Chemistry, Amsterdam University Medical Centers |
| --- | --- |
| Address | Meibergdreef 9  PO Box 22660  1100 DD  Amsterdam  The Netherlands |
| Primary contact name | Joyce Rops |
| Primary contact e-mail address | [j.rops@amsterdamumc.nl](mailto:j.rops@amsterdamumc.nl) |

### Flow cytometry execution

| Name of organization | Laboratory of Experimental Clinical Chemistry, Amsterdam University Medical Centers |
| --- | --- |
| Address | Meibergdreef 9  PO Box 22660  1100 DD  Amsterdam  The Netherlands |
| Contact name | Joyce Rops |
| Contact e-mail address | [j.rops@amsterdamumc.nl](mailto:j.rops@amsterdamumc.nl) |

## Purpose

The goal of this flow cytometry experiment is to investigate whether sample-specific dilution to prevent swarm detection affects the measured EV concentration. Our hypothesis is that applying custom dilution factors to individual samples in a study biases the EV concentration measurements.

## Keywords

Background, Concentration measurement, Dilution, Extracellular vesicles, Flow cytometry

## Experiment variables

The experimental variable is the sample dilution.

## Experiment design and quality controls

All samples were measured using an autosampler, which facilitates subsequent measurements of samples in a 96-well plate. All experiments were performed in flat-bottom 96-wells plates. The entire study involved the experiments shown in Table 1. Details about the used trigger thresholds will be explained later in this document.

*Table 1: Overview of experiments performed for this study. APC: allophycocyanin; CD: cluster of differentiation; FITC: fluorescein isothiocyanate.*

| **Experiment** | **Trigger threshold** | **Used markers** | **Performed on date** |
| --- | --- | --- | --- |
| Lipoprotein spike-in | Side-scatter detector | CD45-APC  CD62p-FITC | 11 feb 2025  10 apr 2025 (replicate) |
| Serial dilutions | Side-scatter detector | CD45-APC  CD62p-FITC | 21 mar 2025  16 apr 2025 (replicate) |
| Lipoprotein spike-in | 638 nm fluorescence detector | CD45-APC | 20 mar 2025  14 apr 2025 (replicate) |
| Serial dilutions | 638 nm fluorescence detector | CD45-APC | 14 apr 2025  17 apr 2025 (replicate) |

The well plates contained buffer-only controls, isotype controls, detergent-lysis controls and labelling-reagents-in-buffer controls. Flow rate, fluorescence, and light scattering calibrations were performed daily.

## Dates

Samples were collected in October 2022 in the Amsterdam University Medical Centers, location Academic Medical Center. Flow cytometry experiments were performed on the aforementioned dates.

## Conclusions

Sample-specific dilution factors affect EV concentration measurements and cause incomparability across samples. Therefore, samples within the same study should be diluted equally.

# Sample details

## Sample description

### Sample source description

Samples were obtained from 20 healthy volunteers, of which 10 are males (median age: 34 years) and 10 are females (median age: 33 years).

### Sample description

Intravenously collected blood.

## Sample collection

Blood was collected in Ethylene Diamine Tetra-acetic Acid (EDTA) tubes.

## Sample storage

The blood was centrifuged for 15 minutes at 2,500 g. Plasma above 10 mm from the pellet was transferred to a clean tube and centrifuged again for 15 minutes at 2,500 g. Plasma above 10 mm from the pellet was collected and pooled in a clean tube. All plasma samples were pooled and aliquots were made. Aliquots were snap frozen in liquid nitrogen and stored at -80 °C until use. The same pooled plasma is used throughout all experiments in this article.

## Sample characteristics

Frozen human plasma samples are expected to contain the following particles: erythrocyte ghosts, EVs, lipoproteins, platelets, precipitated salt crystals, proteins, and complexes of the aforementioned particles [4].

## Sample staining

The optimal incubation time for staining has been determined prior to this study. Unfiltered platelet concentrate was diluted 100x in citrate buffer. 20 µl of the diluted platelet concentrate was incubated with 2.5 µl of CD61-PE (phycoerythrin) or CD9-PE for 15 min, 30 min, 1 hour or 2 hours. After incubation, 1980 µl of DPBS was added. Samples were measured using flow cytometry with scatter-triggering. The results in Figure 1 indicate that the maximum median fluorescence intensity and the maximum fluorescent particle concentration were reached after 2 hours of incubation. Therefore, 2 hours was chosen as the optimal incubation time.


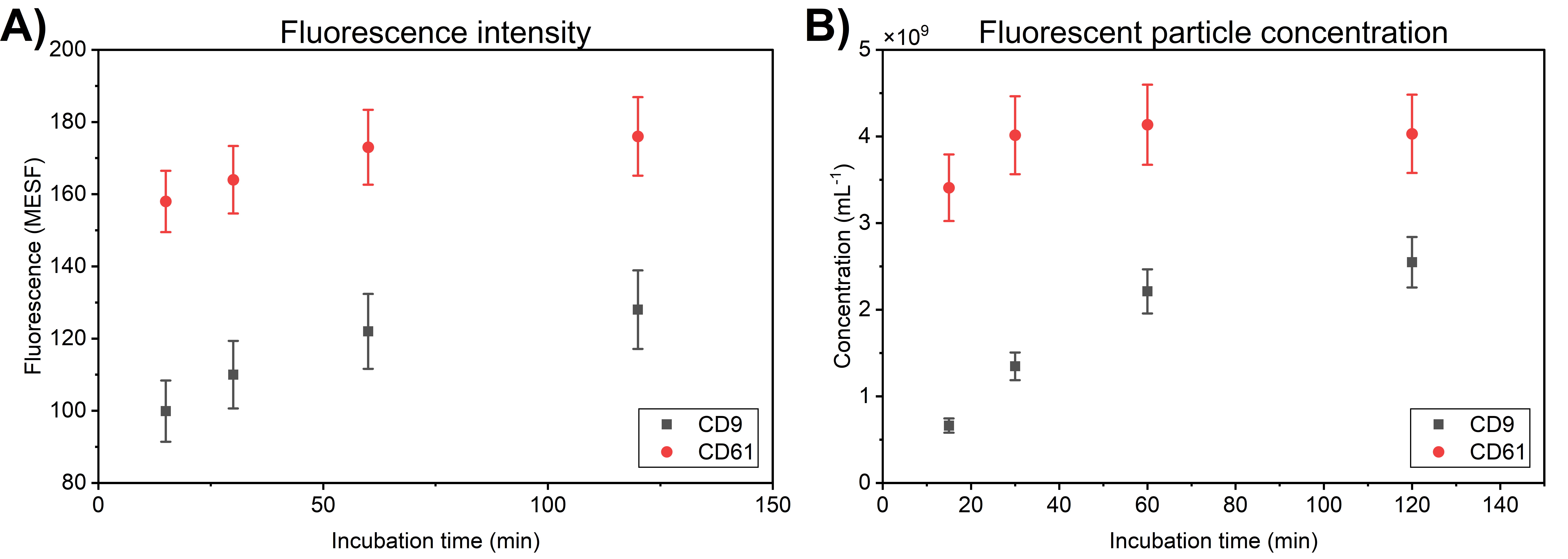


*Figure 1: Optimization of the incubation time for staining. Unfiltered platelet concentrate was diluted and incubated with CD9-PE or CD61-PE for different amounts of time. (A) Median fluorescence intensity versus incubation time shows that the maximum fluorescence intensity was reached at 2 hours. (B) Concentration of fluorescent particles versus incubation time shows that the maximum concentration of fluorescent particles was reached after 1 hour.*

Table 2 shows an overview of the antibodies that were used to stain leukocyte-derived and P-selectin expressing EVs in plasma. Prior to staining, the antibody concentrations were decreased by dilution in DPBS. For each antibody the optimal concentration was determined by titration of (i) CD45 in culture medium of peripheral blood mononuclear cells (PBMC) and (ii) CD62p in plasma. As shown in Figure 2 the optimal concentration was determined by the number of antibody-positive counts (Figures 2A and 2C) and the median intensity of the fluorescent background (Figures 2B and 2D). Please note that the titration of these two antibodies is based on our previous titration procedure, which we have improved in 2023 based on Pink et al. [5]. As a result, for CD45 a suboptimal concentration was used in this study (1.5 µg/mL), leading to a possible underestimation of the number of CD45+ EVs of ~15%. However, this does not affect the study conclusions. The used concentration of CD62p (8.33 µg/mL) was optimal since the saturation was reached and the background was minimized.

During staining of plasma samples in this study, diluted antibodies were centrifuged at 18,890 g for 5 min at 20 °C to remove aggregates. The supernatant minus 10 μL of the starting volume was collected and used for staining. Each sample was stained with the antibodies indicated in Table 1. To stain, 20 μL of pre-staining diluted plasma was incubated with 2.5 μL of each antibody or isotype controls and kept in the dark for 2 h at room temperature. After the incubation, samples were diluted in 200 μL DPBS to decrease background fluorescence from unbound reagents.

## Fluorescence reagents

| **Characteristic**  **measured** | **Analyte** | **Analyte detector** | **Reporter** | **Isotype** | **Clone** | **Stock concentration (µg mL^-1^)** | **Manufacturer** | **Catalog number** | **Lot number** | **Dilution factor** |
| --- | --- | --- | --- | --- | --- | --- | --- | --- | --- | --- |
| Leukocyte common antigen | Human  CD45 | Anti-human CD45 antibody | APC | IgG1 | HI30 | 9 | Biolegend | 304037 | B272158 | 6x |
| P-selectin | Human CD62p | Anti-human CD62p antibody | FITC | IgG1 | CLB-Thromb/6 | 50 | Beckman Coulter | A07790 | 200042 | 6x |

Table 2. Overview of staining reagents. Characteristics being measured, analyte, analyte detector, reporter, isotype, clone, concentration during staining, manufacturer, catalog number and lot number of used staining reagents. The concentration of staining reagents during measurements was 11-fold lower than the concentration during staining. APC: allophycocyanin; CD: cluster of differentiation; FITC: fluorescein isothiocyanate.





*Figure 2: Results from antibody titrations. Optimal antibody concentrations are determined based on the number of antibody-positive events (A and C) and median intensity of the fluorescent background (B and D). (A-B) Titration of CD45-APC in isolated EVs from peripheral blood mononuclear cells. (C-D) Titration of CD62p-FITC in blood plasma. APC: allophycocyanin; CD: cluster of differentiation; FITC: fluorescein isothiocyanate.*

# Flow cytometer

## Model and manufacturer

Apogee A60-Micro, Apogee Flow Systems, Catalogne, Spain.

## Configuration and settings

### Flow rate and acquisition time

Samples were analysed for 120 s at a flow rate of 3.01 μL/min. The A60-Micro is equipped with a syringe pump with volumetric control, and each day the flow rate was calibrated internally (Apogee calibration beads, Apogee Flow Systems).

### Light sources

The flow cytometer has three lasers that illuminate a flow cell. The adjusted powers were 100 mW, 150 mW and 150 mW for the 405-nm, 488-nm, and 638-nm laser, respectively.

### Detectors

Table 3 shows an overview of the detectors used in this study.

| Detector name | Detected property | Voltage (V) | Spectral filter bandwidth (nm) |
| --- | --- | --- | --- |
| 405-SALS | Forward scattered light | 470 |  |
| 405-LALS | Side scattered light | 367 |  |
| 488-Grn | FITC fluorescence | 560 | 525/50 |
| 638D-Red | APC fluorescence | 480 | >650 |

Table 3. Detector name, detected property, voltage and spectral filter bandwidth of the detectors used in this study. APC: allophycocyanin; CD: cluster of differentiation; FITC: fluorescein isothiocyanate.

The detection efficiency Q was determined for both fluorescence detectors using the approach described in [6], as shown in Figure 3. Using the linear fits shown in Figure 3, Q was determined to be 0.06 photoelectrons per MESF for APC and 0.005 photoelectrons per MESF for FITC.





*Figure 3: Analysis of the detection efficiency of the fluorescence detectors used in this article. Q was obtained using these fits following the approach described in [6]. Q = 0.06 photoelectrons per APC-MESF and Q = 0.005 photoelectrons per FITC-MESF. (A-B) Linear fit of the corrected CV versus the square root of the corrected fluorescence intensity. (C-D) Linear fit of the specified fluorescence intensity versus the median fluorescence intensity. APC: allophycocyanin; a.u. = arbitrary unit; CV: coefficient of variation; FITC: fluorescein isothiocyanate; MESF = molecules of equivalent soluble fluorochrome.*

### Trigger detector and threshold

Specified for each experiment in main manuscript.

# Assay controls

We relied on immunofluorescent staining and calibrated signal intensities to confirm that signals originate from leukocyte-derived and P-selectin expressing EVs. All assay controls were measured on the same acquisition settings as the plasma samples. All scatter plots from all controls are publicly available (see Section 5.1).

## Unstained controls

Unstained controls were measured in all experiments, using the same flow cytometer acquisition settings as all other samples. In each dilution- or spike-in series, for each step an unstained control was included. The results of the unstained controls are summarized in Table 4. Representative examples of scatter plots obtained from unstained controls are shown in Figure 4.

*Table 4: Overview of mean number of marker-positive counts found in the unstained controls of each experiment. LP: lipoprotein; CD: Cluster of Differentiation; Fluor: fluorescence; SD = standard deviation.*

| **Experiment** | **Trigger** | **Date** | **CD45+ counts (mean ± SD)** | **Mean CD62p+ counts (mean ± SD)** |
| --- | --- | --- | --- | --- |
| LP spike-in | Scatter | 11-2-2025 | 7.1 ± _3.3 | 19.5 ± 3.8 |
| LP spike-in | Fluor | 20-3-2025 | 71.3 ± 16.6 |  |
| Serial dilution | Scatter | 21-3-2025 | 1.3 ± _2.4 | 1.1 ± 1.7 |
| LP spike-in | Scatter | 10-4-2025 | 8.6 ± _3.6 | 27.9 ± 4.5 |
| LP spike-in | Fluor | 14-4-2025 | 64.0 ± _5.1 |  |
| Serial dilution | Fluor | 14-4-2025 | 62.3 ± _7.6 |  |
| Serial dilution | Scatter | 16-4-2025 | 1.0 ± _1.5 | 1.5 ± 1.9 |
| Serial dilution | Fluor | 17-4-2025 | 58.8 ± _5.3 |  |


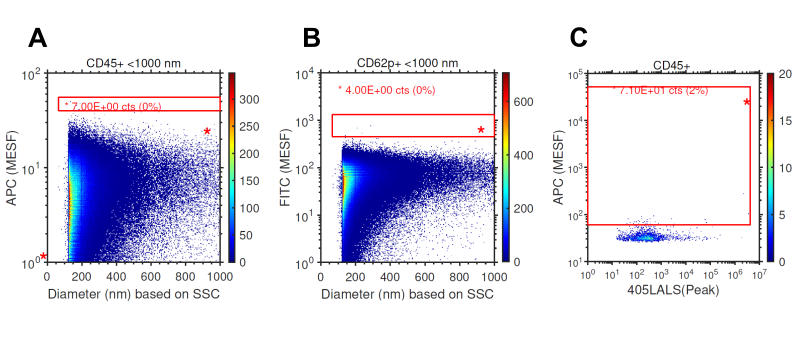


*Figure 4: Representative examples of scatter plots from unstained controls, i.e. measurements of pooled plasma samples of 20 healthy volunteers without staining reagents. The red solid rectangles represent the gates as used for all measurements in the experiment. (A&B) Data represent events that exceeded the side-scattering trigger threshold corresponding to a side-scattering cross section of 3 nm^2^. (C) Data represent events that exceeded the fluorescence trigger threshold corresponding to 32 APC-MESF. APC = allophycocyanin; CD = cluster of differentiation; FITC = fluorescein isothiocyanate; MESF = molecules of equivalent soluble fluorochrome.*

Unstained controls yieled an average of <100 counts in all experiments. The counts found in the experiments using scatter triggering are a factor ~10 lower than the counts found in the experiments using fluorescence triggering. While the amount of counts in the fluorescence-triggered experiments may be acceptable, the difference with the scatter-triggered experiments indicates that further improvement of the fluorescence-triggering protocol may further improve the quality of measurements.

## Reagent-in-buffer controls

Each 96-wells plate included at least one well with reagent-in-buffer control for each used marker. Reagent-in-buffer controls were measured using the same flow cytometer and acquisition settings as all other samples. Results of the reagent-in-buffer controls are summarized in Table 5. Representative examples of scatter plots obtained from reagent-in-buffer controls are shown in Figure 5.

*Table 5: Overview of mean number of marker-positive counts found in the reagent-in-buffer controls of each experiment. LP: lipoprotein; CD: Cluster of Differentiation; Fluor: fluorescence.*

| **Experiment** | **Trigger** | **Date** | **CD45+ counts (mean ± SD)** | **Mean CD62p+ counts (mean ± SD)** |
| --- | --- | --- | --- | --- |
| LP spike-in | Scatter | 11-2-2025 | 23.0 ± 0 (n=1) | 15.0 ± 0 (n=1) |
| LP spike-in | Fluor | 20-3-2025 | 94.0 ± 0 (n=1) |  |
| Serial dilution | Scatter | 21-3-2025 | 20.4 ± 15.7 | 29.1 ± 40.8 |
| LP spike-in | Scatter | 10-4-2025 | 15.3 ± 2.9 | 51.7 ± 42.2 |
| LP spike-in | Fluor | 14-4-2025 | 92.5 ± 27.5 |  |
| Serial dilution | Fluor | 14-4-2025 | 92.5 ± 27.5 |  |
| Serial dilution | Scatter | 16-4-2025 | 17.8 ± 15.2 | 46.8 ± 51.0 |
| Serial dilution | Fluor | 17-4-2025 | 72.5 ± 0.5 |  |

*
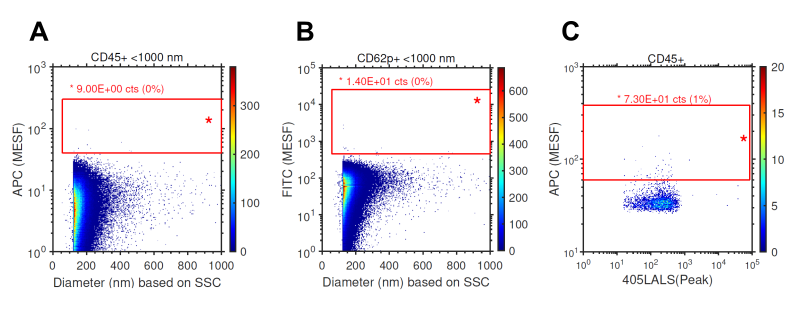
*

*Figure 5: Representative examples of scatter plots from reagent-in-buffer controls. The red solid rectangles represent the gates as used for all measurements in the experiment. (A&B) Data represent events that exceeded the side-scattering trigger threshold corresponding to a side-scattering cross section of 3 nm^2^. (C) Data represent events that exceeded the fluorescence trigger threshold corresponding to 32 APC-MESF. APC = allophycocyanin; CD = cluster of differentiation; FITC = fluorescein isothiocyanate; MESF = molecules of equivalent soluble fluorochrome.*

In the reagents-in-buffer controls we also observed a difference in CD45+ counts between the experiments using scatter-triggering and fluorescence-triggering. Here, it is likely that particles were recorded that were bright enough to exceed the fluorescence trigger threshold, but were too small to exceed the scattering trigger threshold. An example of such particles could be antibody aggregates. Therefore, it is not surprising that this difference between fluorescence-triggered and scatter-triggered controls exists.

## Detergent lysis controls

Each 96-well plate contained at least one well with a plasma sample that contained 1% NP-40 (Merck Life Science, The Netherlands). The sample with detergent was measured at the same flow cytometer and acquisition settings as all other samples. Results of the detergent lysis controls are summarized in Table 6. Representative examples of scatter plots obtained from detergent lysis controls are shown in Figure 6.

*Table 6: Overview of mean number of marker-positive counts found in the detergent lysis controls of each experiment. LP: lipoprotein; CD: Cluster of Differentiation; Fluor: fluorescence.*

| **Experiment** | **Trigger** | **Date** | **CD45+ counts (mean ± SD)** | **CD62p+ counts (mean ± SD)** |
| --- | --- | --- | --- | --- |
| LP spike-in | Scatter | 11-2-2025 | 20 ± 0 (n=1) | 22 ± 0 (n=1) |
| LP spike-in | Fluor | 20-3-2025 | 301.5 ± 188.5 |  |
| Serial dilution | Scatter | 21-3-2025 | 25.0 ± 4.8 | 33.3 ± 25.8 |
| LP spike-in | Scatter | 10-4-2025 | 44.3 ± 14.9 | 70.7 ± 23.6 |
| LP spike-in | Fluor | 14-4-2025 | 133.5 ± 5.2 |  |
| Serial dilution | Fluor | 14-4-2025 | 135.0 ± 17.0 |  |
| Serial dilution | Scatter | 16-4-2025 | 23.3 ± 2.7 | 46.3 ± 30.5 |
| Serial dilution | Fluor | 17-4-2025 | 185.3 ± 59.0 |  |


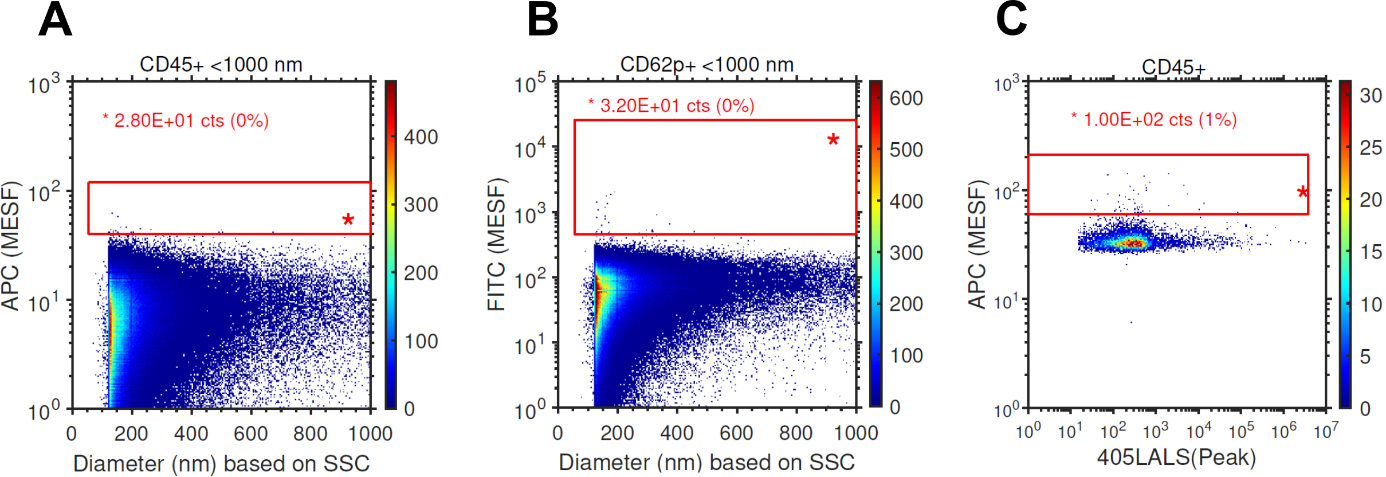


*Figure 6: Representative examples of scatter plots from detergent lysis controls. The red solid rectangles represent the gates as used for all measurements in the experiment. (A&B) Data represent events that exceeded the side-scattering trigger threshold corresponding to a side-scattering cross section of 3 nm^2^. (C) Data represent events that exceeded the fluorescence trigger threshold corresponding to 32 APC-MESF. APC = allophycocyanin; CD = cluster of differentiation; FITC = fluorescein isothiocyanate; MESF = molecules of equivalent soluble fluorochrome.*

In the detergent lysis controls of some fluorescence-triggered experiments we observed an unexpected population that contributed to the CD45+ counts in the high scattering-signal region, as illustrated by the example in Figure 7. In scatter-triggered experiments gates are used to only count particles <1000 nm. However, because (i) our size calibration is based on side-scattering signals and (ii) there is swarm detection on the side-scattering detector in the fluorescence-triggered experiments, we were unable to perform size calibration on this data. As the population appears in the high side-scattering region (~ >2·10^5^ a.u.) we believe this is a population we would not observe in our scatter-triggered experiments where they would be gated out. We only observe this population in detergent lysis controls, or in wells directly following the detergent lysis control (spill-over). Therefore, we suspect these events are associated with microbubbles or micron-sized micelles originating from the detergent.

In the scatter-triggered experiments the detergent lysis controls yielded on average <100 counts, as expected.


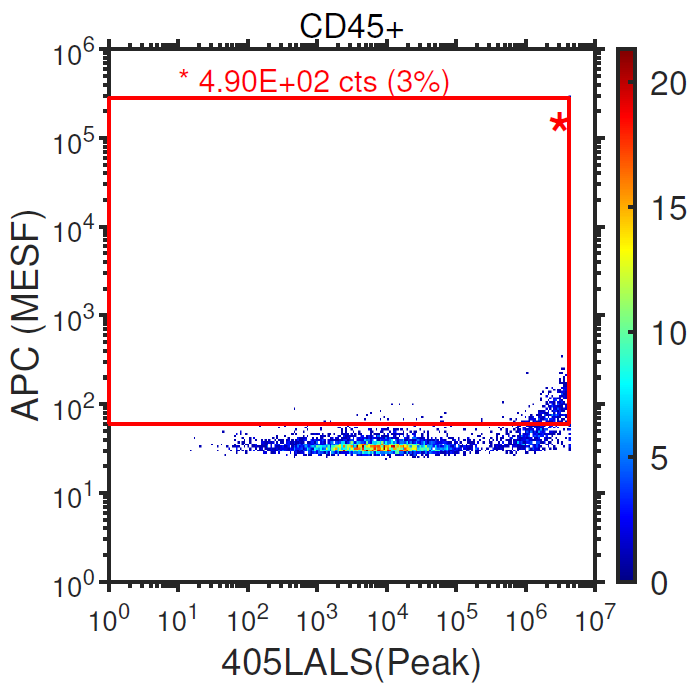


*Figure 7: Example of a scatterplot of a detergent lysis control from a fluorescence-triggered experiment where an unexpected population appears at scattering signals ~ >2·10^5^ arbitrary units. These particles are suspected to originate from the detergent itself, rather than from the sample or other reagents.*

## Buffer-only controls

Each 96-wells plate included at least one well with buffer-only control for each used marker, which was measured at the same flow cytometer and acquisition settings as all other samples. Results of the buffer-only controls are summarized in Table 7. Representative examples of scatter plots obtained from buffer only controls are shown in Figure 8.

*Table 7: Overview of mean number of marker-positive counts found in the buffer-only controls of each experiment. LP: lipoprotein; CD: Cluster of Differentiation; Fluor: fluorescence.*

| **Experiment** | **Trigger** | **Date** | **CD45+ counts (mean ± SD)** | **Mean CD62p+ counts (mean ± SD)** |
| --- | --- | --- | --- | --- |
| LP spike-in | Scatter | 11-2-2025 | 0.5 ± 0.5 | 20.5 ± 26.0 |
| LP spike-in | Fluor | 20-3-2025 | 62.5 ± 10.5 |  |
| Serial dilution | Scatter | 21-3-2025 | 1.5 ± 1.6 | 6.8 ± 9.7 |
| LP spike-in | Scatter | 10-4-2025 | 0 ± 0 | 6.7 ± 6.0 |
| LP spike-in | Fluor | 14-4-2025 | 58.4 ± 6.2 |  |
| Serial dilution | Fluor | 14-4-2025 | 60.2 ± 4.9 |  |
| Serial dilution | Scatter | 16-4-2025 | 0.9 ± 1.3 | 5.1 ± 7.4 |
| Serial dilution | Fluor | 17-4-2025 | 87.0 ± 59.7 |  |


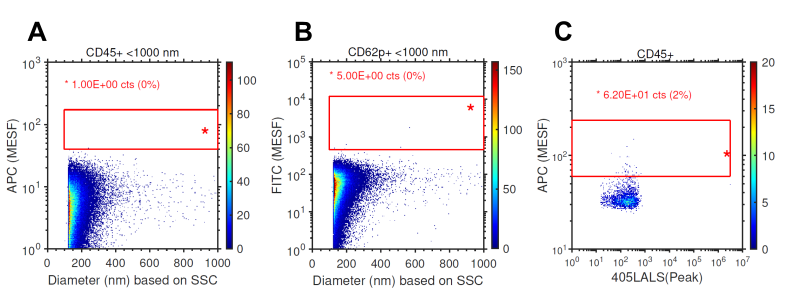


*Figure 8: Representative examples of scatter plots from buffer only controls. The red solid rectangles represent the gates as used for all measurements in the experiment. (A&B) Data represent events that exceeded the side-scattering trigger threshold corresponding to a side-scattering cross section of 3 nm^2^. (C) Data represent events that exceeded the fluorescence trigger threshold corresponding to 32 APC-MESF. APC = allophycocyanin; CD = cluster of differentiation; FITC = fluorescein isothiocyanate; MESF = molecules of equivalent soluble fluorochrome.*

In all experiments the buffer-only controls yielded on average <100 counts with a narrow standard deviation. The only exception is the experiment on 17-4-2025, where the buffer-only control was done right after the detergent-lysis control. Spill-over from the detergent-lysis control resulted in an unexpected population at high side-scattering levels, similar to the population illustrated in the example in Figure 7.

## Isotype controls

Isotype controls were measured in all experiments. In each dilution- or spike-in series, for each step an isotype control was included. Results of the isotype controls are summarized in Table 8. Representative examples of scatter plots obtained from isotype controls are shown in Figure 9.

*Table 8: Overview of mean number of marker-positive counts found in the isotype controls of each experiment. APC = allophycocyanin; LP: lipoprotein; FITC = fluorescein isothiocyanate; Fluor: fluorescence.*

| **Experiment** | **Trigger** | **Date** | **APC+ counts (mean ± SD)** | **Mean FITC+ counts (mean ± SD)** |
| --- | --- | --- | --- | --- |
| LP spike-in | Scatter | 11-2-2025 | 44.6 ± 90.5 | 93.9 ± 32.6 |
| LP spike-in | Fluor | 20-3-2025 | 257.2 ± 399.8 |  |
| Serial dilution | Scatter | 21-3-2025 | 1.3 ± 2.4 | 15.3 ± 7.3 |
| LP spike-in | Scatter | 10-4-2025 | 13.6 ± 4.2 | 132.4 ± 28.3 |
| LP spike-in | Fluor | 14-4-2025 | 66.7 ± 19.4 |  |
| Serial dilution | Fluor | 14-4-2025 | 81.8 ± 23.2 |  |
| Serial dilution | Scatter | 16-4-2025 | 2.7 ± 2.6 | 10.9 ± 5.6 |
| Serial dilution | Fluor | 17-4-2025 | 72.7 ± 10.6 |  |


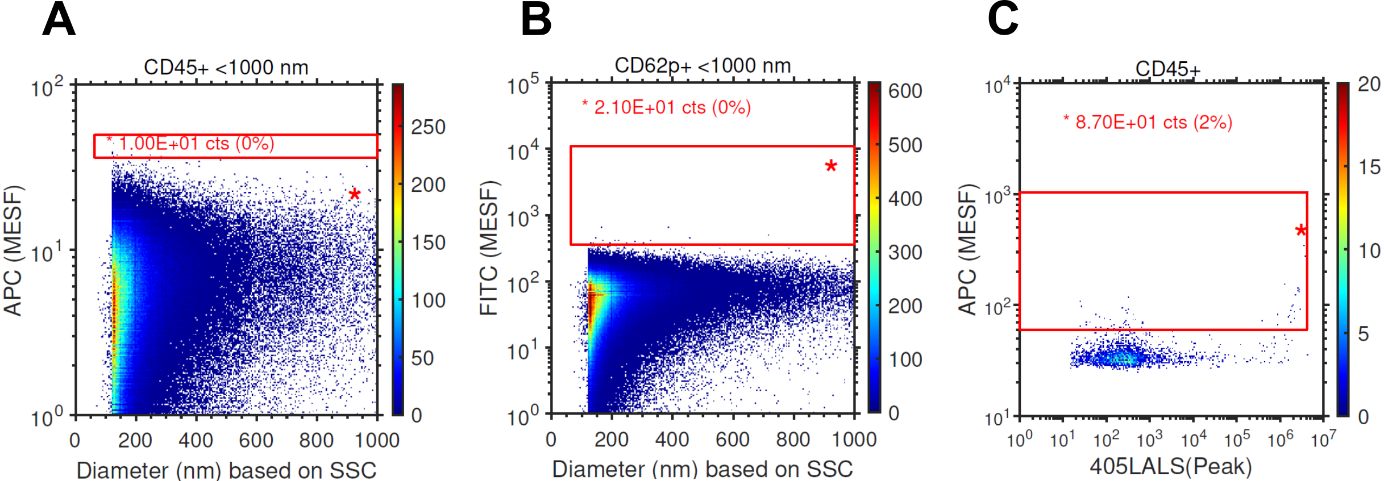


*Figure 9: Representative examples of scatter plots from isotype controls. The red solid rectangles represent the gates as used for all measurements in the experiment. (A&B) Data represent events that exceeded the side-scattering trigger threshold corresponding to a side-scattering cross section of 3 nm^2^. (C) Data represent events that exceeded the fluorescence trigger threshold corresponding to 32 APC-MESF. APC = allophycocyanin; CD = cluster of differentiation; FITC = fluorescein isothiocyanate; MESF = molecules of equivalent soluble fluorochrome.*

In the lipoprotein spike-in experiments measured by scatter-triggering, FITC+ counts of IgG1 isotype controls were generally higher than CD62p+ EV counts. In the main manuscript we established that only fluorescent background counts have been measured for CD62p. This suggests that CD62p generates less background than the applied IgG1 control. The IgG1 isotype control is not a perfect control, because it is a different type of antibody from a different clone. Therefore, it is possible that the used IgG1 control is more ‘sticky’ than the used CD62p antibody. Another explanation could be that the IgG1 antibody concentration provided by the manufacturer was lower than the actual IgG1 antibody concentration. Since only CD62p+ background counts were measured in these experiments, the high IgG1 counts do not affect our conclusions.

In the experiment of 11-2-2025 we observed an unexpected population in the IgG1-APC control in one condition. Since the population was only visible in the measurement of one well, we expect that a contamination was present in this well. Therefore we omitted the data from this well during analysis.

In the experiment of 20-3-2025, high IgG1 counts were observed in one condition due to spill-over from the detergent lysis control. This explains the high mean and standard deviation given in Table 8. No further action was taken.

Otherwise, isotype controls yielded consistently low counts, as expected.

# Data analysis

To automatically apply calibrations, determine and apply gates, generate reports with scatterplots and generate data summaries, we developed and applied custom-build software (MATLAB R2020b, Mathworks, USA).

## Data sharing

Data are publicly available on Figshare: 10.6084/m9.figshare.31073530.

## Compensation details

No compensation was applied because no fluorophore combinations were used that have overlapping emission spectra.

## Calibrations

### Flow rate

At the start of each measurement day, the flow rate was validated internally using ApoCal beads (Apogee Flow Systems, Northwood, United Kingdom). The Apogee A60-Micro is equipped with a syringe pump with volumetric control. A flow rate of 3.01 µl/min was assumed for all measurements.

### Fluorescence calibration

Calibration of the fluorescence detectors from arbitrary units (a.u.) to molecules of equivalent soluble fluorochrome (MESF) was accomplished using commercially available 2-µm APC quantification beads (lot 2364-19, custom-order, Becton Dickinson Biosciences), and 2-µm FITC quantification beads (lot 2364-85, custom-order, Becton Dickinson Biosciences). Figure 10A and 10B show the 10-base logarithm of the MESF intensities for the MESF beads versus the 10-base logarithm of the measured median fluorescence intensity of each bead population. The data are fitted with a linear function. These fluorescence calibrations were used to assign MESF values for APC and PE to rainbow beads (SPHERO^TM^ Rainbow calibration particles, 8 peaks, 3.0-3.4 µm, lot EAP01, Spherotech). In turn, the rainbow beads, which are hard dyed beads with long-term stability, were used to apply fluorescence calibrations on a daily basis. Figure 10C and 10D show the 10-base logarithm of the assigned MESF intensities for rainbow beads versus the 10-base logarithm of the measured median fluorescence intensity of each bead population. The data are fitted with a linear function. For each measured plasma sample, we added fluorescent intensities in MESF units to the flow cytometry data files using the following equation:

| $\text{I(MESF)}={10}^{a\cdot\log_{10} \text{I(a.u.)}+b}$ | Equation 1 |
| --- | --- |

where I is the fluorescence intensity, and *a* and *b* are the slope and the intercept of the linear fits in Figure 10. Calibration of the MESF beads was only done once before the start of this study, results in Figures 10A and 10B. Cross-calibration using the rainbow beads was done every measurement day. An example of the cross-calibration data is shown in Figures 10C and 10D, and the exact fit values of each measurement day are shown in Table 9.


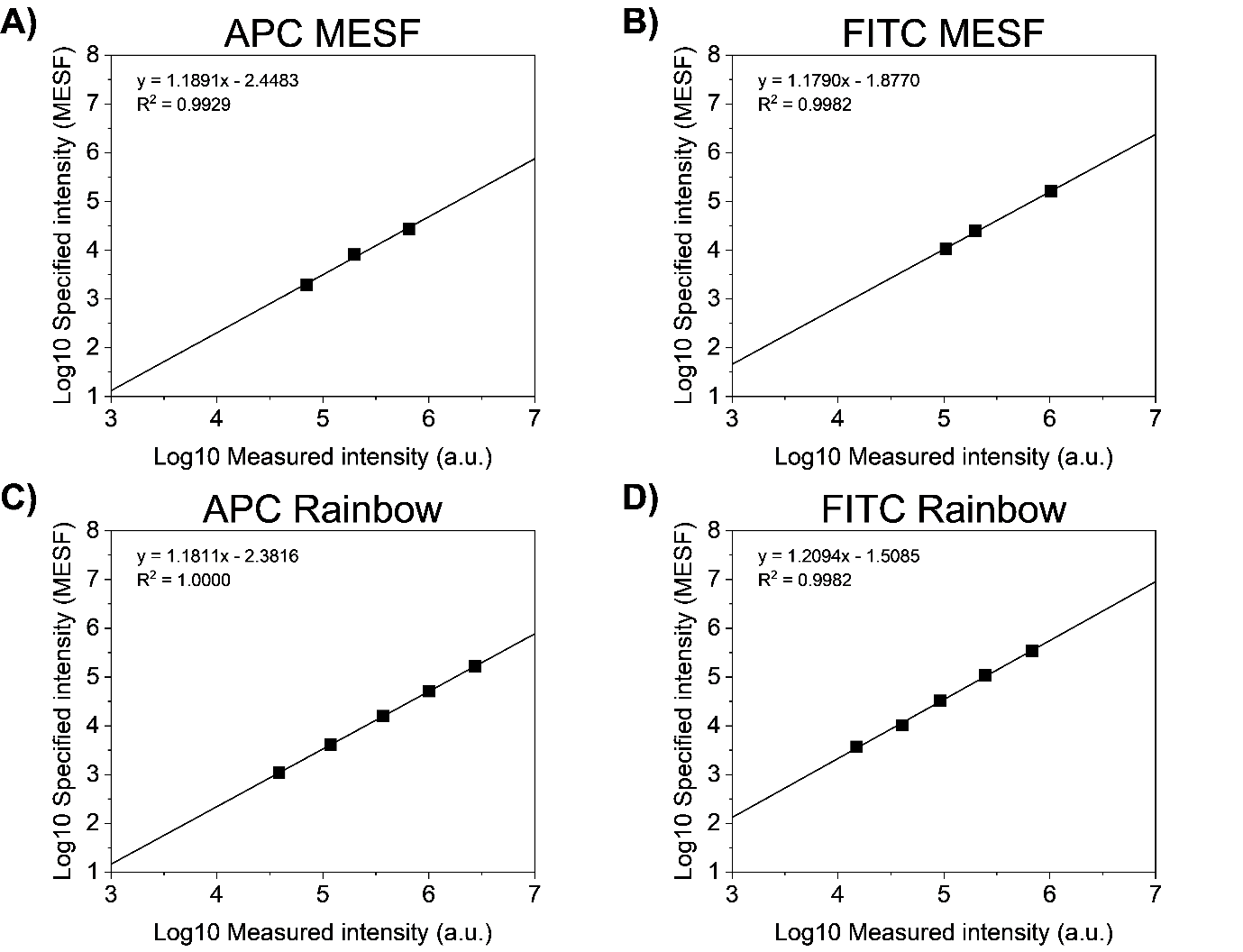


Figure 10: Calibration of the fluorescence detectors from arbitrary units (a.u.) to molecules of equivalent soluble fluorochrome (MESF). Logarithmic MESF versus logarithmic median fluorescence intensity for (A) allophycocyanin (APC), and (B) fluorescein isothiocyanate (FITC). Furthermore, the 10-base logarithm of the assigned MESF intensities for rainbow beads versus 10-base logarithm of the measured median fluorescence intensity of each bead population are shown for APC (C), and FITC (D). Data (symbols) are fitted with a linear function (line).

*Table 9: Overview of linear fit values of Equation 1 to the data from the rainbow bead cross-calibration. Calibration was performed by plotting the 10-base logarithm of the assigned MESF intensities for rainbow beads versus 10-base logarithm of the measured median fluorescence intensity of each bead population, like shown in Figure 1C and 1D.*

|  | **APC** | | **FITC** | |
| --- | --- | --- | --- | --- |
| **Date** | **Fit** | **R^2^** | **Fit** | **R^2^** |
| 11-2-2025 | y = 1.1811x – 2.3816 | 1.0000 | y = 1.2094x – 1.5085 | 0.9982 |
| 20-3-2025 | y = 1.1821x – 2.3765 | 1.0000 | - | - |
| 21-3-2025 | y = 1.1823x – 2.3800 | 1.0000 | y = 1.1793x – 1.7693 | 1.0000 |
| 10-4-2025 | y = 1.1825x – 2.3875 | 1.0000 | y =1.1747x – 1.7333 | 1.0000 |
| 14-4-2025 | y = 1.1821x – 2.3853 | 1.0000 | - | - |
| 16-4-2025 | y = 1.1821x – 2.3799 | 1.0000 | y = 1.1803 – 1.7693 | 1.000 |
| 17-4-2025 | y = 1.1818x – 2.3749 | 1.0000 | - | - |

### Light scattering calibration

#### Rosetta Calibration


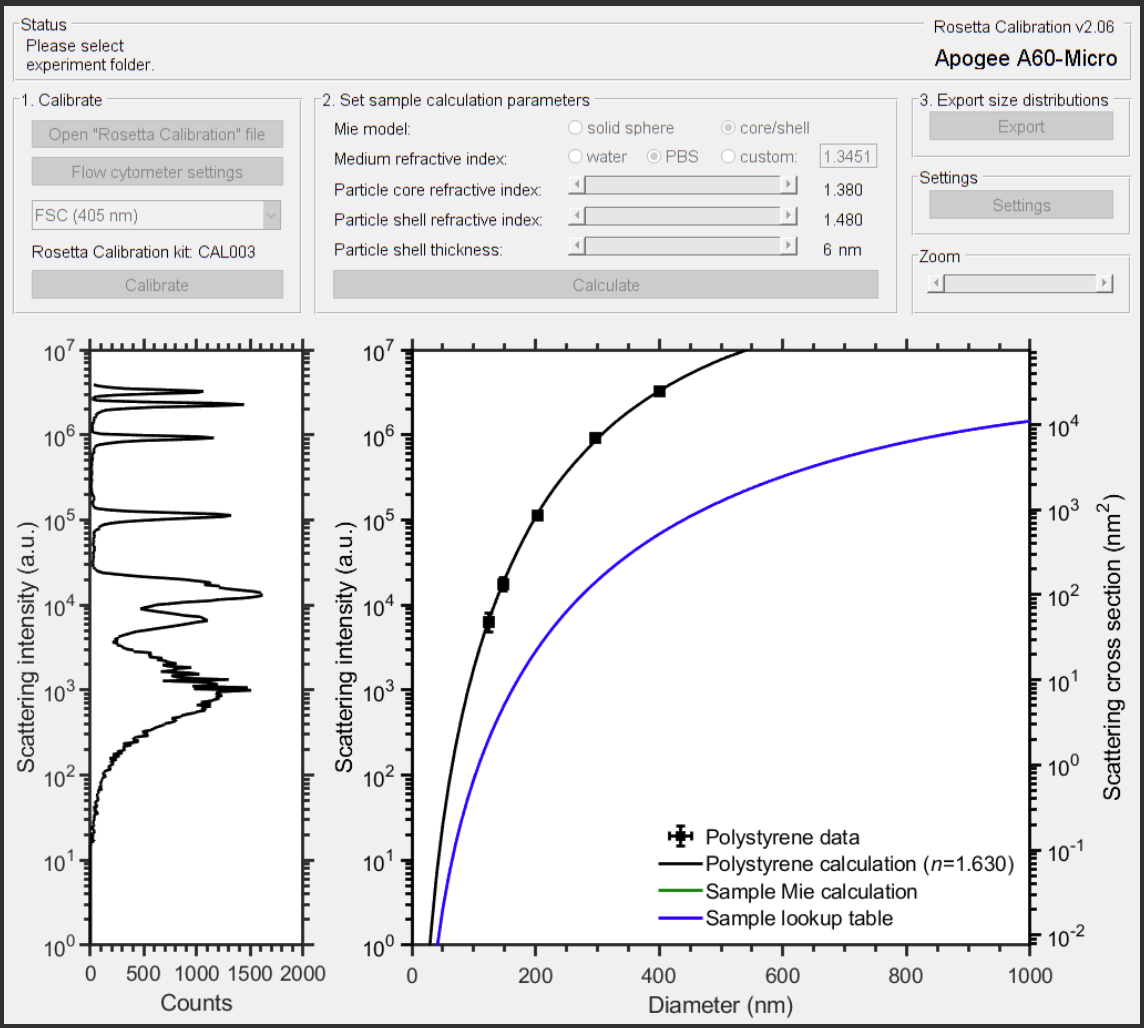
Rosetta Calibration (v2.06, Exometry, The Netherlands) was used to relate the side scattering intensities measured at a wavelength of 405 nm to the effective scattering cross sections^^[[1]](#footnote-1)^^ and optical diameter^^[[2]](#footnote-2)^^ of cells. Cells are modelled as core-shell particles with a core refractive index of 1.38, a shell refractive index of 1.48, and a shell thickness of 6 nm. Figure 11 shows a print screen of the light scatter calibration of one measurement day. This calibration was performed daily. Table 10 shows the scaling factor relating the raw side scattering signals (a.u.) to the side scattering cross section (nm^2^) for each measurement day.

Figure 11. Side scattering calibration of the Apogee A60-Micro. To relate scatter to the diameter of cells, cells are modelled as core-shell particles with a core refractive index of 1.38, a shell refractive index of 1.48, and a shell thickness of 6 nm.

*Table 10: Used scaling factors to convert raw side-scattering signals (SSC) in arbitrary unit to side-scattering cross sections (SSCS) in nm^2^ per measurement day. Scaling factor is only given for experiments where size-calibration was applied. LP: lipoprotein; Fluor: fluorescence.*

| **Experiment** | **Trigger** | **Date** | **Scaling factor SSC to SSCS** |
| --- | --- | --- | --- |
| LP spike-in | Scatter | 11-2-2025 | 301.3234638 |
| LP spike-in | Fluor | 20-3-2025 |  |
| Serial dilution | Scatter | 21-3-2025 | 263.5203075 |
| LP spike-in | Scatter | 10-4-2025 | 273.4240406 |
| LP spike-in | Fluor | 14-4-2025 |  |
| Serial dilution | Fluor | 14-4-2025 |  |
| Serial dilution | Scatter | 16-4-2025 | 266.4556774 |
| Serial dilution | Fluor | 17-4-2025 |  |

## Gate description and boundaries

Gates values were automatically determined and applied using custom-build software (MATLAB R2020b) [8]. All MESF gates have been specified in the main manuscript and are summarized in Table 11.

PDF files with scatterplots of all applied gates are publicly available at Figshare: DOI: 10.6084/m9.figshare.31073530. Representative examples are given for all experiments in Figures 12 and 13.

*Table 11: Overview of the applied fluorescence gates in each experiment. APC = allophycocyanin; LP: lipoprotein; CD: Cluster of Differentiation; FITC = fluorescein isothiocyanate; Fluor: fluorescence; MESF = molecules of equivalent soluble fluorochrome.*

| **Experiment** | **Trigger** | **Date** | **APC gate** | **FITC gate** |
| --- | --- | --- | --- | --- |
| LP spike-in | Scatter | 11-2-2025 | 38 MESF | 949 MESF |
| LP spike-in | Fluor | 20-3-2025 | 60 MESF |  |
| Serial dilution | Scatter | 21-3-2025 | 40 MESF | 450 MESF |
| LP spike-in | Scatter | 10-4-2025 | 36 MESF | 356 MESF |
| LP spike-in | Fluor | 14-4-2025 | 60 MESF |  |
| Serial dilution | Fluor | 14-4-2025 | 60 MESF |  |
| Serial dilution | Scatter | 16-4-2025 | 40 MESF | 450 MESF |
| Serial dilution | Fluor | 17-4-2025 | 60 MESF |  |

**
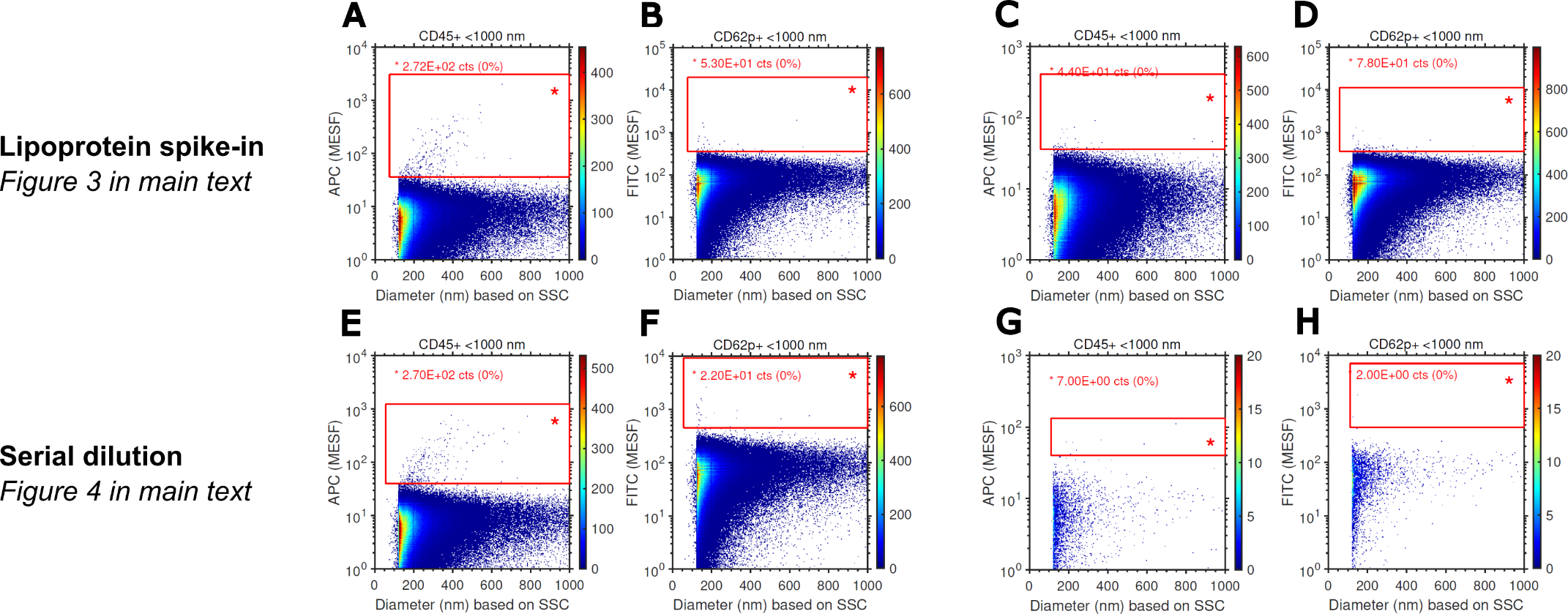
**

*Figure 12: Representative scatter plots of measurements at low and high dilution using scatter triggering. Data represent events that exceeded the side-scattering trigger threshold corresponding to a side-scattering cross section of 3 nm^2^. The red solid rectangle represents the applied gate. The number displayed in red inside the scatterplot is the number of events inside the gate, i.e. the detected total antibody-positive particle number. Applied pre-staining dilutions: (A & B) 4.5·10^1^; (C & D) 6.4·10^4^; (E & F) 4.5·10^1^ (G & H) 8.9·10^3^. A post-staining dilution of 11.25 was applied to all samples. APC = allophycocyanin; CD = cluster of differentiation; FITC = fluorescein isothiocyanate; MESF = molecules of equivalent soluble fluorochrome.*

**
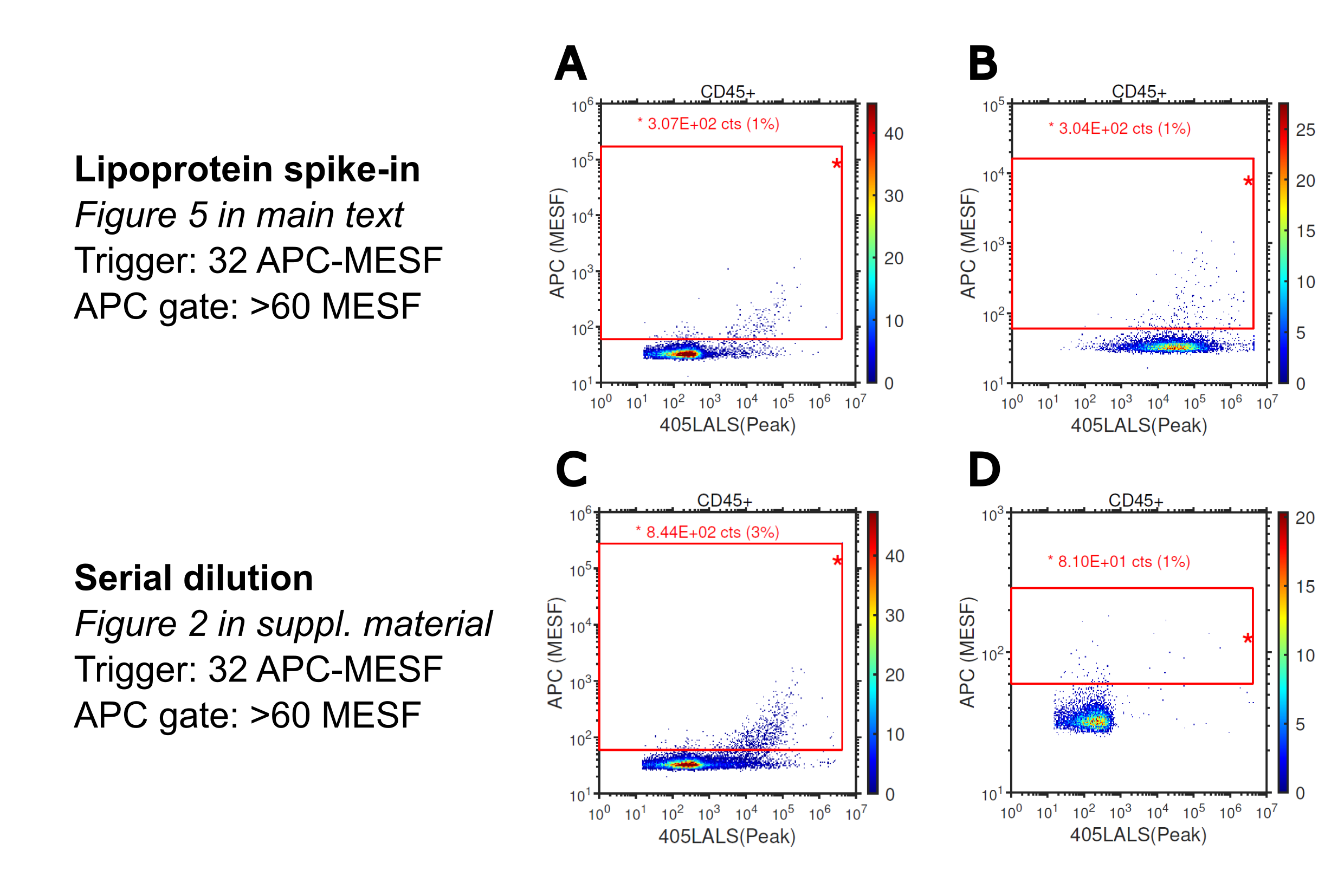
**

*Figure 13: Representative scatterplots of samples measured using fluorescence triggering. Data represent events that exceeded the fluorescence threshold as specified on the left. (A) Unspiked sample with a total particle concentration of 7.3·10^10^ mL^-1^, measured with a pre-staining dilution of 45 and a post-staining dilution of 11.25. (B) Plasma sample with spiked-in lipoproteins with a total particle concentration of 1.0·1013 mL^-1^, measured with a pre-staining dilution of 45 and a post-staining dilution of 11.25. (C) Plasma sample with a total particle concentration of 7.3·10^10^ mL^-1^, measured with a pre-staining dilution of 10 and a post-staining dilution of 11.25. (D) Plasma sample with a total particle concentration of 7.3·10^10^ mL^-1^, measured with a pre-staining dilution of 889 and a post-staining dilution of 11.25. APC = allophycocyanin; CD = cluster of differentiation; EVs = extracellular vesicles; FITC = fluorescein isothiocyanate; MESF = molecules of equivalent soluble fluorochrome.*

# References

[1] Théry C, Witwer KW, Aikawa E, Alcaraz MJ, Anderson JD, Andriantsitohaina R, Antoniou A, Arab T, Archer F, Atkin-Smith GK, others. Minimal information for studies of extracellular vesicles 2018 (MISEV2018): a position statement of the International Society for Extracellular Vesicles and update of the MISEV2014 guidelines. J Extracell Vesicles 2018; 7: 1535750.

[2] Lee JA, Spidlen J, Boyce K, Cai J, Crosbie N, Dalphin M, Furlong J, Gasparetto M, Goldberg M, Goralczyk EM, others. MIFlowCyt: the minimum information about a Flow Cytometry Experiment. Cytometry Part A Wiley Online Library; 2008; 73: 926–30.

[3] Welsh JA, van der Pol E, Arkesteijn GJA, Bremer M, Brisson A, Coumans F, Dignat-George F, Duggan E, Ghiran I, Giebel B, Görgens A, Hendrix A, Lacroix R, Lannigan J, Libregts SFWM, Lozano-Andrés E, Morales-Kastresana A, Robert S, de Rond L, Tertel T, et al. MIFlowCyt-EV: a framework for standardized reporting of extracellular vesicle flow cytometry experiments. J Extracell Vesicles 2020; 9: 1713526.

[4] Bettin B, Gasecka A, Li B, Dhondt B, Hendrix A, Nieuwland R, van der Pol E. Removal of platelets from blood plasma to improve the quality of extracellular vesicle research. J Thromb Haemost Wiley; 2022; 20: 2679–85.

[5] Pink D, Basu A, Wong M, Pham D, Valencia J, Triana V, et al. Antibody titrations are critical for microflow cytometric analysis of extracellular vesicles. Cytometry Part A. 2023 Aug 1;103(8):670–83. doi:10.1002/cyto.a.24733

[6] Chase ES, Hoffman RA. Resolution of Dimly Fluorescent Particles: A Practical Measure of Fluorescence Sensitivity. Cytometry. 1998;33:267–79. doi:10.1002/(SICI)1097-0320(19981001)33:2<267::AID-CYTO24>3.0.CO;2-R

[7] Welsh JA, Arkesteijn GJA, Bremer M, Cimorelli M, Dignat-George F, Giebel B, Görgens A, Hendrix A, Kuiper M, Lacroix R, others. A compendium of single extracellular vesicle flow cytometry. J Extracell Vesicles Wiley Online Library; 2023; 12: e12299.

[8] Gankema AAF, Li B, Nieuwland R, Pol E van der. Automated fluorescence gating and size determination reduce variation in measured concentration of extracellular vesicles by flow cytometry. Cytometry Part A; 2022 Dec 1;101(12):1049–56.

1. The effective scattering cross section is a hypothetical area of a particle that incoming light must impinge in order to be scattered towards the lens. The calibrated effective scattering cross section axis is independent of refractive index assumptions, but depends on the illumination wavelength and collection angles of the flow cytometer [7]. [↑](#footnote-ref-1)
2. The optical diameter equals the physical diameter of a particle when (1) the particle is spherical and (2) the particle has the same refractive index distribution as assumed in the model. [↑](#footnote-ref-2)
